# Mechanism of lesion verification by the human XPD helicase in nucleotide excision repair

**DOI:** 10.1093/nar/gkac496

**Published:** 2022-06-17

**Authors:** Iwen Fu, Hong Mu, Nicholas E Geacintov, Suse Broyde

**Affiliations:** Department of Biology, New York University, 100 Washington Square East, New York, NY 10003, USA; Department of Biology, New York University, 100 Washington Square East, New York, NY 10003, USA; Department of Chemistry, New York University, 100 Washington Square East, New York, NY 10003, USA; Department of Biology, New York University, 100 Washington Square East, New York, NY 10003, USA

## Abstract

In nucleotide excision repair (NER), the xeroderma pigmentosum D helicase (XPD) scans DNA searching for bulky lesions, stalls when encountering such damage to verify its presence, and allows repair to proceed. Structural studies have shown XPD bound to its single-stranded DNA substrate, but molecular and dynamic characterization of how XPD translocates on undamaged DNA and how it stalls to verify lesions remains poorly understood. Here, we have performed extensive all-atom MD simulations of human XPD bound to undamaged and damaged ssDNA, containing a mutagenic pyrimidine (6−4) pyrimidone UV photoproduct (6−4PP), near the XPD pore entrance. We characterize how XPD responds to the presence of the DNA lesion, delineating the atomistic-scale mechanism that it utilizes to discriminate between damaged and undamaged nucleotides. We identify key amino acid residues, including FeS residues R112, R196, H135, K128, Arch residues E377 and R380, and ATPase lobe 1 residues 215−221, that are involved in damage verification and show how movements of Arch and ATPase lobe 1 domains relative to the FeS domain modulate these interactions. These structural and dynamic molecular depictions of XPD helicase activity with unmodified DNA and its inhibition by the lesion elucidate how the lesion is verified by inducing XPD stalling.

## Graphical Abstract

**Graphical Abstract:**
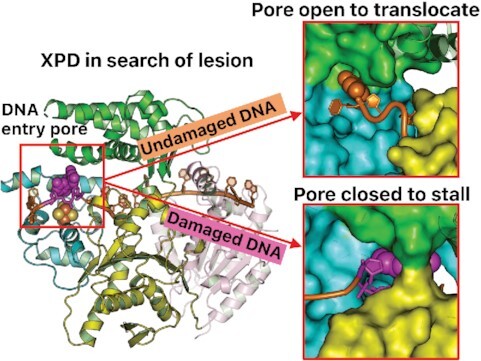
MD simulations of XPD–ssDNA complexes show that XPD opens its pore to allow undamaged DNA to translocate and narrows its pore to stall the lesion.

## INTRODUCTION

Global genomic nucleotide excision repair (GG–NER) is an important and highly conserved DNA repair mechanism for a wide range of bulky DNA lesions generated by exogenous sources such as solar UV and chemical carcinogens (1–9). Absorption of UV sunlight causes two adjacent pyrimidines to be covalently crosslinked, generating highly prevalent and mutagenic UV-induced photolesions (6,10–13). Among these, the pyrimidine (6−4) pyrimidone photoproduct (6−4PP) is notable because it is well repaired in cells (4,12,14,15), while the cyclobutane pyrimidine dimer (CPD) that is generated in several-fold greater amount than 6−4PP (16,17) is poorly repaired (18–22). Failure in eliminating the photolesions leads to a proclivity for genetic disorders characterized by UV hypersensitivity, development of malignant skin cancers, and accelerated aging (7,8,12,23–25).

The GG–NER pathway entails initial damage recognition, damage verification, and damage dual incision, followed by gap filling (1,3,4,7,9,26–28). GG–NER for bulky and distorting lesions is initiated by a versatile damage sensor, the XPC protein complex, facilitated by the UV-damaged DNA-binding protein (UV-DDB). XPC recognizes the local helical distortions imposed by the lesion and then binds the undamaged strand opposite the lesion-containing one, to form an XPC–DNA complex with a small bubble of ∼2−3 nucleotides (27–30). This complex recruits the ten-subunit protein complex transcription factor IIH (TFIIH) that is required for both transcription initiation and NER (1,31–37). A recent cryo-EM structure (38) of the yeast TFIIH/Rad4-Rad23-Rad33/DNA complex provides structural understanding of TFIIH recruitment to the lesion by the XPC–DNA complex. In NER, TFIIH unwinds the DNA around the lesion. The TFIIH core (seven-subunits excluding the kinase module) promotes the opening of the DNA around the lesion, facilitated by XPA (1,7,34,37,39–42). A cryo-EM structure (39) of the human core TFIIH bound to a forked DNA substrate and to XPA reveals that the XPB translocase binds the duplex DNA that is locked in place by XPA, and the XPD helicase binds the single-strand 3′-DNA extension. XPB further opens a bubble around the damage, facilitating delivery of the damaged single-stranded DNA to XPD; then, XPD tracks along the ssDNA in a 5′−3′ direction to verify the damage (40,42–44). This is followed by dual incision of the damaged strand by endonucleases XPF and XPG that eliminate nucleotides on the 5′ and the 3′ side of the lesion, respectively, leaving a ∼24−32 nt single-stranded oligomer. The gap is filled by one or more DNA polymerases that use the undamaged partner nucleotides as a template, and then the DNA ends are rejoined by a DNA ligase to complete NER.

While TFIIH is essential for both transcription initiation and NER, XPD plays a much less crucial role in transcription initiation, and its 5′−3′ helicase activity is exclusively important for NER (45–47). Inherited mutations in the XPD gene are found in patients with the genetic diseases xeroderma pigmentosum (XP), trichothiodystrophy (TTD) and Cockayne syndrome (24,48–51); the latter is associated with transcription coupled nucleotide excision repair. Structural and biochemical studies of XPD either in the apo state or in complex with ssDNA have revealed details about the conserved nature of the XPD fold and the binding path of the ssDNA in XPD (39,46,52–57) (reviewed in ref. (58)). Although archaeal XPD homologs are isolated as monomers with no stable interactions with other proteins while eukaryotic XPD is part of TFIIH, all structural studies of XPD reveal a very similar four-domain organization. It consists of an Arch domain and an FeS domain as well as two helicase/ATPase lobe motor domains, which are commonly referred to as (HD1 and HD2)/(ATPase lobe 1 and ATPase lobe 2). Note that we used the term “ATPase lobes 1 and 2” in our study, as they are referred to in the cryo-EM structure of the XPD−ssDNA complex with PBD ID 6RO4 (39) that is the basis of our models. In other cases, we retain the terminology “HD1 and HD2” utilized in the relevant literature. The FeS domain contains a 4Fe4S cluster coordinated by four sulfhydryl cysteine amino acids; the oxidation state of the 4Fe4S cluster is a mixture of Fe2+/Fe3+, whose redox potential is within physiological range (59). Notably, the HD1 domain together with the FeS and Arch domains generate a remarkable pore-like feature with a size that is sufficient to accommodate ssDNA and that is altered upon DNA binding and ATP binding/hydrolysis. The translocated ssDNA path goes along the HD1 and HD2 domains, and then reaches across the XPD pore with its 3′-end. A number of studies have suggested a role for this entry pore in helicase activity, damage verification and NER efficiency (47,51,53,54,56,60–62).

As XPD searches for lesions, it verifies their presence. Several lines of evidence have established XPD as a main sensor for verifying the presence of bulky DNA lesions in NER (40,41,43,52,63). Structural studies have characterized XPD interactions with the ssDNA (39,57), identifying the entire DNA binding path, the entry pore and the adjacent lesion recognition pocket (1,54). Despite these extensive structural, biochemical and biophysical analyses, XPD bound with lesion-containing ssDNA that contacts the entire binding path, with dynamic information for both damaged and undamaged DNA that contains the extended DNA outside the pore, remain unexplored. Molecular and dynamic delineations of how XPD translocates on the undamaged DNA and blocks DNA lesions near the entry pore presented here offer new insights on the key process of lesion verification.

Our present study is focused on the UV-induced 6−4PP lesion as a model of an abundant bulky lesion that is rigid and very distorting, due to the 6−4 linkage with nearly perpendicular orientation of its two thymine rings. We employed molecular modeling of human XPD bound to ssDNA containing the 6−4PP lesion placed right *outside* and *within* the XPD pore and carried out extensive all-atom MD simulations of ∼3.5 μs to characterize the interactions of XPD with the lesion. A remarkable finding in our MD study is that the unmodified nucleotides near the XPD pore undergo a one-nucleotide backbone translocation in a 3′→5′ direction while the 6−4PP lesion near the pore is nearly immobilized. We note that the 5′−3′ translocation of XPD along the DNA is equivalent to the DNA 3′−5′ translocation within the XPD. We characterize the XPD-DNA interactions near the pore and identify the important residues, including the FeS residues R112, R196, H135, K128, the Arch residues E377 and R380, and the ATPase lobe 1 helix with residues 215−221, that form extensive interactions with the lesion and thereby immobilize it. By contrast, these residues manifest a dynamic network of interactions with the unmodified nucleotides near the pore, allowing the undamaged DNA to translocate. Our results highlight the roles of these key residues in facilitating XPD discrimination of damaged from undamaged DNA.

## MATERIALS AND METHODS

### Initial models

#### XPD-unmod-noExtendedDNA

We wished to understand how the XPD protein verifies the presence of the lesion near the DNA entry pore. We started with the cryo-EM structure of the human core TFIIH-XPA-DNA complex with 3.6 Å resolution in PBD ID 6RO4 (39) (Supplementary Figures S1A−B and S2A). In this cryo-EM structure, the sequence of eleven nucleotides of ssDNA (11nt-ssDNA) bound to XPD is 5′-AATGAGCACAT-3′, from Chain B with residue numbers 32−42. The 3′-end of the ssDNA is accommodated within the XPD pore, which is formed by the Arch, FeS and ATPase lobe 1 domains. Notably, the 3′-end nucleotide dT (3′dT) is positioned at the edge of the entry pore, where the Arch residue R380’s side chain is in close contact with the ATPase lobe 1 helix, comprised of residues 215−221; as a result, it forms a closed conformation of the pore (Supplementary Figures S1A and S2A). The nucleotides outside this pore are not visible in this cryo-EM structure. Also, in the Arch domain there is a mobile region, comprised of residues 273−325, which is not visible in the cryo-EM structure; this region would clash with the DNA at the XPD pore, as revealed in the apo-TFIIH structures with PDB ID 6NMI (37) and PDB ID 5OF4 (55), where the residues form a plug to occupy the DNA entry pore of XPD. In our current study, we excluded this mobile region (residues 273−325) by capping residues 272 and 326 with a C-terminal N-methyl amide and an N-terminal acetyl group, respectively, which is the standard MD procedure for treating the missing residues in cryo-EM or crystal structure. This yields the XPD bound to the 11nt-ssDNA without the DNA extension to its 3′-end (namely XPD-unmod-noExtendedDNA); this model is utilized in the subsequent MD simulations of the XPD in complex with the damaged and undamaged DNA.

#### XPD-lesionOUT and XPD-lesionIN

**Figure 1.**
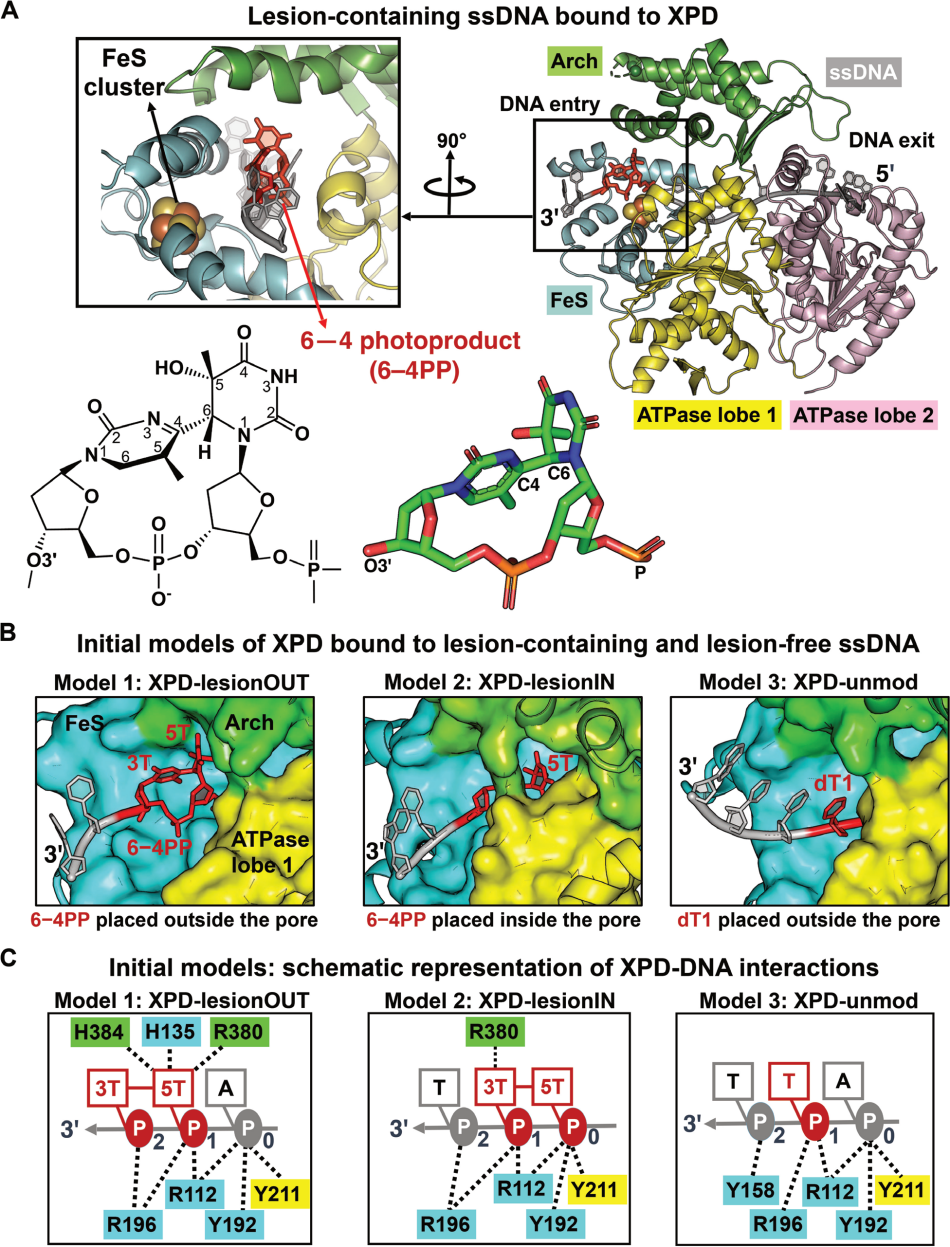
Initial structures of XPD complex with the lesion-containing and lesion-free ssDNA and the interactions of the XPD–ssDNA near the entry pore. (**A**) Initial structure of XPD complex with the lesion (red stick)-containing ssDNA (grey). This initial structure is the first snapshot in the production run of the MD simulation. The XPD protein includes four domains: the Arch, FeS, ATPase lobe 1 and lobe 2 domains, which are color coded and labelled according to constituent subunits. XPD has a central tunnel that accommodates ssDNA with its 3′- and 5′-end extended toward the entry and exit pore, respectively. Inset box showing a view into the DNA entry pore, formed by the FeS, Arch, and ATPase lobe 1 domains. The iron-sulfur cluster (FeS cluster) adjacent to the DNA entry pore is indicated as orange and yellow spheres, respectively. The lesion is the pyrimidine (6−4) pyrimidone photoproduct (6−4PP). The chemical and crystal structures of the 6−4PP lesion, which is taken from a Rad4-Rad23 structure bound to a 24-bp DNA containing a 6−4PP lesion (PDB ID 6CFI (64)) are shown here; the structure shows that the two thymine rings are linked by a single C6−C4 covalent bond and are roughly perpendicular. (**B**) Zoom-in view of the DNA entry pore depicts the initial structures of the XPD complex with lesion-containing and lesion-free ssDNA. Note that initial structures for each model are the first snapshot in the production run in the MD simulations. Full details concerning the preparation of the initial models for the MD simulations of XPD–ssDNA complex are given in Materials and Methods and Supplementary Figures S1 and S2. (**C**) Schematic representation of interactions of XPD with the nucleotides at positions 0 to 2 near the entry pore for each initial structure as described in (B). The 5T and 3T of the lesion indicate the pyrimidine and pyrimidone of 6−4PP, respectively. These XPD-DNA interactions mainly involve R112, Y192, R196 and Y211 that anchor the DNA backbone moieties.

To investigate how XPD responds to the presence of the bulky lesion, we built two lesion-containing models in which the single-stranded DNA contains a 6−4PP lesion at its 3′-end near the entry pore (Figure 1). The 6−4PP structure is based on that in the crystal structure of a damaged DNA duplex bound to Rad4-Rad23 with PDB ID 6CFI (64). In the first lesion-containing model, the 3′-end nucleotide dT (3′dT) of 11nt-ssDNA in PDB ID 6RO4 (39) was replaced with a 6−4PP lesion with its bases 5T and 3T positioned *outside* the edge of the XPD pore (Model 1 in Supplementary Figure S1C); two more nucleotides d(TG) were then added to the 3′ side of the lesion using standard B-DNA in Discovery Studio 2.5 (Accelrys Software Inc.), producing a final model of the XPD complex with 6−4PP lesion-containing ssDNA (namely XPD-lesionOUT) (see Model 1 in Figure 1B). For the second lesion-containing model, the last two nucleotides d(TA) at the 3′-end of the ssDNA in PDB ID 6RO4 were replaced with the 6−4PP by aligning the bases 5T and 3T of the lesion with dT and dA in PBD ID 6RO4, respectively (see Model 2 in Supplementary Figure S1C). Thus, the lesion, with its 5T and 3T bases, is positioned *inside* the edge of the XPD pore. Two additional nucleotides d(TG) in B-form were added to the 3′ side of the lesion, giving the final model of XPD-lesionIN (Model 2 in Figure 1B).

#### XPD-unmod

We also generated an XPD complex with lesion-free ssDNA. For this lesion-free model, the 3′dT in PDB ID 6RO4 (39) was replaced with extended B-DNA with sequence d(TTTG) (Model 3 in Supplementary Figure S1C), yielding an initial model for XPD-unmod (see Model 3 in Figure 1B). Note that in this initial model, we retained the position of the 3′dT phosphate moiety in PDB ID 6RO4 (39) and then manually reoriented the base and sugar of dT1 *outside* the pore and *toward* the ATPase lobe 1 helix (residues 215−221); thus, the dT1 base can stack with its 3′-nucleotide (dT2) in B-form without causing steric clashes with the Arch residues R380 and H384 (Supplementary Figure S2B). Most importantly, the dT1 base of the extended DNA is shifted outside the pore, creating an unoccupied space within the pore (zoom-in view in Supplementary Figure S2B). Therefore, this initial model of XPD-unmod permits investigation of translocation, while the cryo-EM structure of XPD−ssDNA complex with PBD ID 6RO4 (39), the model of XPD-unmod-noExtendedDNA, is not a translocation-capable one—it neither includes the incoming DNA extended outside the entry pore since it is not resolved in the structure, nor has enough space within the pore for one additional nucleotide (Supplementary Figure S2A).

### MD simulations

All XPD–ssDNA systems were subject to energy minimization, equilibration, and ∼2−3.5 μs production runs of MD simulations using AMBER18 (Case, *et al.*, AMBER 2018, University of California, San Francisco, 2018). The XPD–ssDNA systems in XPD-unmod, XPD-lesionOUT and XPD-lesionIN had 3.5 μs production runs, while additional eight ∼2 μs production runs (8 × 2 μs) for the undamaged DNA in XPD-unmod were performed to further explore the conformational space of the flexible DNA extension. In addition, we have carried out a ∼2 μs MD simulation of the original cryo-EM structure with PDB ID 64RO4 (39) of XPD bound to 11nt-ssDNA *without* the DNA extension to its 3′-end (XPD-unmod-noExtendedDNA) as a control, to investigate the impact of the flexible DNA extension on the structure and dynamics of the XPD pore residues. All MD simulations began with minimization and equilibration steps that would mitigate any strains that might be present in the initial models, ensuring the systems are equilibrated during the production step. Details concerning force field, the routine MD protocol and structural analyses are given in the Supplementary Methods.

## RESULTS

### Overview

We wished to elucidate how the XPD protein verifies the presence of a bulky lesion within the single-stranded DNA. The cryo-EM structure of the human TFIIH/XPA/DNA complex (PDB ID 6RO4), reported by the Cramer laboratory (39), provides an excellent model of XPD within TFIIH bound to the single-strand 3′-DNA extension. In this XPD–ssDNA complex, the ssDNA threads through a narrow channel within XPD and spans along two ATPase lobe domains with its 3′- and 5′-end bound to ATPase lobe 1 and lobe 2 domains, respectively (Supplementary Figure S1A). Notably, the ATPase lobe 1 together with the Arch and the FeS domains form a pore that is spanned by the 3′-end of the ssDNA. This entry pore has been implicated as a lesion scanning region and could potentially detect different types of lesions (1,54,60). We noted that the Arch domain has an extremely mobile region (so called ‘plug’, comprised of residues 273−325), which is not visible in this cryo-EM structure of XPD–ssDNA complex with PDB ID 6RO4 (39) that was the basis of our models; thus, we excluded this Arch plug in our simulations using the standard capping procedure (details see Materials and Methods). In close vicinity to the pore, there is a pocket, comprised of residues Y192 and R196 together with aromatic residues Y158, F161, and F193, that acts in verifying the presence of the lesion on the DNA (Supplementary Figure S1B) (39,60). Thus, we modelled an UV-induced photolesion, 6−4PP (64) at two locations near this entry pore (Figure 1): translationally positioned right outside the entry pore and within the pore, generating two models of the lesion-containing ssDNA bound to XPD, XPD-lesionOUT and XPD-lesionIN, respectively (see Models 1 and 2 in Figure 1B). For each of these cases, we then carried out ∼3.5 μs MD simulation of the XPD bound to the lesion-containing ssDNA.

**Figure 2.**
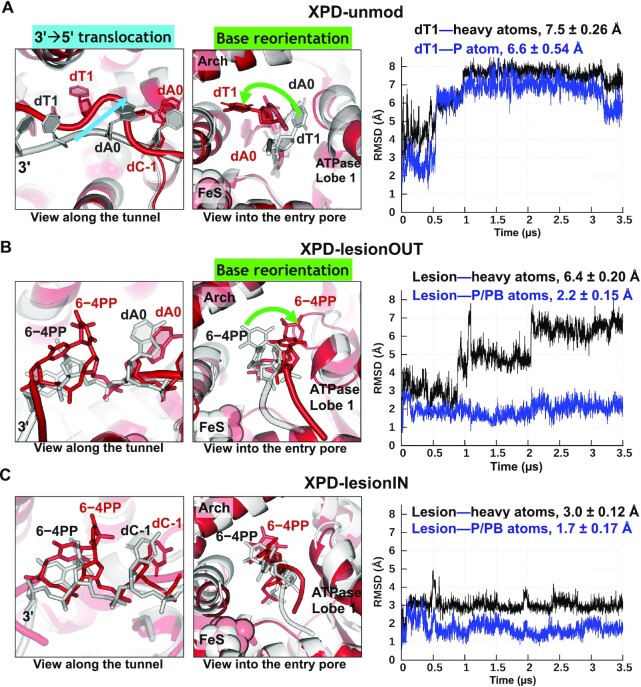
Superposition of the most representative structure (red) from the equilibrated ensemble with the initial structure (grey) and RMSDs of the DNA near the XPD pore for each XPD complex, revealing a one-nucleotide 3′→5′ backbone translocation and base reorientation in the unmodified dT1, while the backbone translocation is absent in the lesion-containing XPDs. (**A**) In XPD-unmod, during the equilibrated state, the dT1 exhibits a large displacement from its initial position within XPD (with heavy atom RMSD of 7.5 ± 0.26 Å). This displacement is dominated by its backbone 3′→5′ movement, as viewed along the entry pore (with P atom RMSD of 6.6 ± 0.54 Å), as well as the base reorientation. A view into the entry pore shows that the base of dT1 is reoriented from the ATPase lobe 1 domain to the interface between the Arch and the FeS domains. (**B**) In XPD-lesionOUT, during the equilibrated state, the 6−4PP lesion reveals a large deviation from its initial conformation (with heavy atom RMSD of 6.5 ± 0.20 Å). This deviation is mostly due to the reorientation of the modified bases that transit from state 1 (0−1 μs) to state 2 (1−2 μs), and to the equilibrated state (2−3 .5 μs) (Supplementary MovieS5); while one-nucleotide backbone translocation is not achieved as revealed in the P atom RMSD of 2.2 ± 0.15 Å. (**C**) In XPD-lesionIN, the lesion retains a stable conformation after ∼0.6 μs with heavy-atom RMSDs of 3.0 ± 0.12 Å, indicating that both backbone and base orientations are similar to the initial conformations. The backbone is nearly immobilized with P atom RMSD of 1.7 ± 0.17 Å. For each XPD–ssDNA complex, we computed the time-dependent heavy atom and backbone P atom RMSDs of the 6−4PP lesion in the lesion-containing XPDs as well as the corresponding RMSDs of the dT1 in the lesion-free XPD.

We also wished to understand how the XPD pore residues respond differently to the damaged and undamaged DNA, and we therefore, we performed a ∼3.5 μs MD simulation of an XPD complex with lesion-free ssDNA (XPD-unmod) (Model 3 in Figure 1B). To further sample the conformations of lesion-free DNA, we carried out additional eight ∼2 μs simulations for a total of 9 independent simulations for XPD-unmod. These nine simulations showed that the undamaged dT1 initially positioned outside the pore displays three types of behavior; 6 of 9 simulations revealed that it is blocked from translocating via entrapment of its base; 2 of 9 simulations showed that it is flipped into the unoccupied space within the pore through base translocation; in 1 of the 9 simulations (revealed in the 3.5 μs simulation), a 3′→5′ backbone translocation was observed, mainly with the backbone entering the unoccupied space within the pore (Figure 2A, Supplementary MovieS1).

The remarkable 3′→5′ backbone translocation that occurred mainly during the 0−1 μs interval of the 3.5 μs production run prior to equilibration, although least prevalent, suggests a possible structural and dynamic mechanism for the biologically important helicase translocation process that takes place during the ATP binding/hydrolysis cycle (65,66). In the presence of the lesion, however, the XPD immobilizes the bulky 6−4PP near the pore (Figure 2B and Supplementary MovieS2 for XPD-lesionOUT, Figure 2C and Supplementary MovieS3 for XPD-lesionIN). A comparison of our three 3.5 μs simulations elucidated how XPD can verify the lesion by preventing translocation, utilizing the trajectories between 2.0 and 3.5 μs for the ensemble analyses that capture the properties of the equilibrated states in these three XPD–ssDNA cases, and how 3′→5′ backbone translocation of unmodified DNA may occur by utilizing the 0−1 μs interval of the 3.5 μs production run prior to equilibration in the lesion-free XPD. Altogether, our MD study provides molecular and dynamic insights on how XPD can distinguish the bulky DNA lesion from the undamaged DNA and elucidates the roles of key XPD residues in fostering lesion stalling and translocating unmodified DNA.

### XPD retains a correctly folded pore when binding to ssDNA regardless of the presence or absence of the lesion

To examine the overall stability of the XPD pore that binds the ssDNA during the 3.5 μs MD simulations (XPD-unmod, XPD-lesionIN and XPD-lesionOUT), we calculated the Cα atom RMSDs of the XPD pore, which is formed by the FeS, Arch, and ATPase lobe 1 domains (Supplementary Figure S3). Overall, our results revealed that after 1.5 μs for these three XPD cases, the XPD pore retains a stable and correctly folded conformation with RMSDs ∼1.4–2.6 Å from the cryo-EM XPD pore structure (39). We note that in XPD-lesionIN, the XPD fold deviates most from its initial state (with RMSDs of ∼2.6 Å) and is the most dynamic during the 0.3–1.5 μs interval (Supplementary Figure S3C). This mobility of the XPD fold is mainly dominated by the FeS helix, containing residues 128–138 (Supplementary Figure S4), which is partially flipped away (particularly with residues 128–133) from the DNA and disordered; however, beginning at ∼1.5 μs, the side chains of residues K128, D129 and D131 are flipped back toward the DNA, greatly reducing the subsequent dynamics of the XPD fold (Supplementary MovieS4).

### The undamaged DNA near the XPD pore displays a one-nucleotide backbone translocation in the 3′−5′ direction while this backbone motion is absent for the lesion

To gain insight on the structural and dynamic properties of the DNA near the entry pore, we computed the RMSDs of heavy-atoms and the backbone P atom of the 6−4PP lesion in the lesion-containing XPDs, as well as the corresponding RMSDs of the undamaged dT1 in the lesion-free XPD while superimposing the XPD pore (Figure 2); these results revealed that the DNA reaches an equilibrated state after ∼1, 2 and 0.5 μs for XPD-unmod, XPD-lesionOUT and lesion-lesionIN, respectively. Altogether, our MD simulations show that the DNA near the entry pore reaches an equilibrated state after ∼2 μs (Figure 2) and the XPD pore stabilizes after 1.5 μs (Supplementary Figure S3). Hence, we utilized the simulations between 2.0 and 3.5 μs for the ensemble analyses to capture the properties of the equilibrated states for these three XPD–ssDNA cases.

Strikingly, when equilibrated, the undamaged dT1 deviates greatly from its initial position. This large deviation is mainly due to its backbone displacement with P atom RMSD of 6.6 ± 0.54 Å (Figure 2A); this is in the range of the nucleotide phosphate to phosphate distance of ∼7 Å in B-DNA, indicating that the backbone of this undamaged DNA is translocated one-nucleotide (1nt) in the 3′−5′ direction. However, in the lesion-containing XPDs, such a backbone translocation is not achieved for the lesion since the P atom RMSD of 2.2 ± 0.15 Å for XPD-lesionOUT (Figure 2B) and 1.7 ± 0.17 Å for XPD-lesionIN (Figure 2C) are much less than the value of 7 Å, required for the 1nt backbone translocation; thus, the backbone translocation is absent for the damaged DNA. Note that the mean values of the P atom RMSDs represent the displacement of the P atom at the equilibrated state from its initial position and therefore reveal whether the backbone of the nucleotide is translocated within the entry pore. Additionally, the undamaged dT1 exhibits higher mobility than the lesion, as reflected in the greater standard deviations in the P atom RMSD, indicating that the XPD grips more tightly the backbone of the lesion than the undamaged dT1.

### An open and flexible entry pore allows the undamaged DNA to be translocated, while a closed and less flexible pore obstructs lesion translocation

#### XPD generates a notable gap between the Arch and the ATPase lobe 1 domains in the presence of the undamaged DNA at the entry pore

The XPD entry pore is formed by the Arch, FeS, and ATPase lobe 1 domains. In the initial structure of each XPD complex, right at the edge of the entry pore, the side chain of R380 points toward D219 and L220 of the ATPase lobe 1 domain; the closest distance of ∼2.8 Å between the Arch residue R380 and these two lobe 1 residues D219 and L220 produces a closed state of the pore (Supplementary Figure S5B). In the case of XPD-unmod, early in the simulation (within tens of nanoseconds), we observed a notable enlargement of the distance between the Arch helix containing R380 and the ATPase lobe 1 helix (with residues 215−221, referred to as ‘the ATPase lobe 1 helix’ hereafter); this is reflected in the increase in the Cα(R380)−Cα(L220) distance by ∼7 Å compared to its initial value (Supplementary Figure S5A), indicating an opposite motion between these two domains. Furthermore, R380 has its side chain pointing away from the ATPase lobe 1, as reflected in the large increase in the shortest distance between R380 and the ATPase lobe 1 helix by ∼8 Å compared to the initial frame (Supplementary Figure S5B). These rearrangements cause a remarkable gap between these two domains, leading to an open state of the pore (Supplementary Figure S5C), which is dynamic and persists throughout the simulation. However, this notable gap is negligible in the lesion-containing XPDs and nearly absent when the lesion is within the pore in the case of XPD-lesionIN (Supplementary Figure S5D−E). We note that in all these three cases, we observed opposite movement of the Arch domain with respect to the ATPase lobe 1 domain in the very early stage, reflected in the increased Cα(R380)−Cα(L220) distance (Supplementary Figure S5A). This mainly stems from the presence of the extended DNA that would interfere with the XPD pore residues. We have additionally performed a ∼2 μs MD simulation of the original cryo-EM structure with PBD ID 6RO4 (39) of XPD bound to 11nt-ssDNA *without* the DNA extension to its 3′-end (XPD-unmod-noExtendedDNA); this simulation shows that the last two nucleotides dT1 and dA0 from the 3′-end are bound stably within the entry pore (Supplementary Figure S6), in accord with the cryo-EM structure. We also observed that without the DNA extension protruding outside the pore, the opposite relative movement between the Arch and the ATPase lobe 1 domains is minimal/negligible (Supplementary Figure S6B), highlighting the important impact of the biologically relevant and flexible incoming DNA in the vicinity of the entry pore on the structure and dynamics of the XPD pore residues.

#### XPD narrows its pore width to restrain the modified bases

Right at the entry pore, there are four helices from the domains encircling the 3′-end DNA in cryo-EM structure with PBD ID 6RO4 (39) (Supplementary Figure S7A). Thus, we monitored the distance Cα(H135)−Cα(L220) as an approximate indicator of the pore width. In this cryo-EM structure (39), this distance is 16.7 Å (Supplementary Figure S7A). We found that in the case of XPD-unmod, the distance is significantly enlarged with ensemble average value of 20.3 ± 0.53 Å, while for lesion-containing XPD, the pore width is either retained at the value of 16.3 ± 0.16 Å in XPD-lesionOUT or narrowed with value of 14.8 ± 0.25 Å in XPD-lesionIN (Supplementary Figure S7B). Notably, the great enlargement of the pore in XPD-unmod is mainly facilitated by the opening of the ATPase lobe 1 helix that is tilted away from the DNA, while the FeS helix (residues 128–138) retains its position/orientation. On the other hand, for the lesion-containing XPDs, this ATPase lobe 1 helix retains its helical position/orientation. However, in XPD-lesionIN, H135 of the FeS helix moves toward the lesion and forms stable close contacts with the methyl group of the lesion 3T base via methyl-π interactions (Supplementary Figure S4C); thus, this FeS helix is tilted slightly toward the lesion or the ATPase lobe 1 domain, narrowing the pore. Two aligned structures, one from XPD-lesionIN and another from XPD-unmod, illustrate that when encountering a lesion within the pore, both ATPase lobe 1 and FeS helices are closer to the lesion than when the DNA is undamaged (Supplementary Figure S8).

A remarkable finding in our simulation of XPD-unmod is that the distance Cα(H135)−Cα(L220) is related to the P atom RMSDs of the backbone DNA near the entry. As revealed in Supplementary Figure S7C, there is a rapid jump in P atom RMSDs of the unmodified nucleotide at ∼0.5 μs, revealing that the nucleotide backbone is being translocated into the pore; this is concomitant with the increased distance of Cα(H135)−Cα(L220) at this time frame (Supplementary Figure S7B). This correlation supports the concept that this distance is a good indicator of the response of the XPD pore to the unmodified or damaged DNA: for the lesion-containing XPDs, the XPD pore is narrower and less dynamic, as reflected in the shorter distance and smaller standard deviations, respectively, than in the lesion-free XPD. Thus, when encountering a lesion near the entry, particularly within the pore (XPD-lesionIN), the XPD forms a tighter and more rigid pore compared to the lesion-free XPD to grip the lesion. On the other hand, when an undamaged DNA is near the entry, the XPD pore becomes more flexible so it can be enlarged to allow the DNA to pass through. We note that the distance Cα(H135)−Cα(L220) only involves the FeS helix and ATPase lobe 1 helix (Supplementary Figure S7A) and contains no motion information about the Arch domain. Furthermore, the dynamics of this distance represents only that of two Cα atoms and does not reflect the overall mobility of the XPD pore domains (Supplementary Figure S3). The relative motion of the Arch domain with respect to the FeS domains is further estimated from the Cα RMSDs of the Arch domain, after fitting the FeS domain Cα atoms to the initial structure for each XPD–ssDNA case (Supplementary Figure S7D); in XPD-unmod, the Arch domain, with respect to the FeS domain, deviates the most from its initial structure at ∼0.5 μs when the DNA backbone is being translocated into the pore and exhibits higher mobility than in the lesion-containing XPDs, as reflected in the greater RMSD fluctuations. These results indicate that with undamaged DNA, the Arch domain is more mobile with respect to the FeS domain than in the presence of the lesion.

### In the lesion-free XPD, the nucleotides enter the pore via one nucleotide backbone 3′→5′ translocation and base reorientation, driven by dynamic interactions between DNA and XPD pore

**Figure 3.**
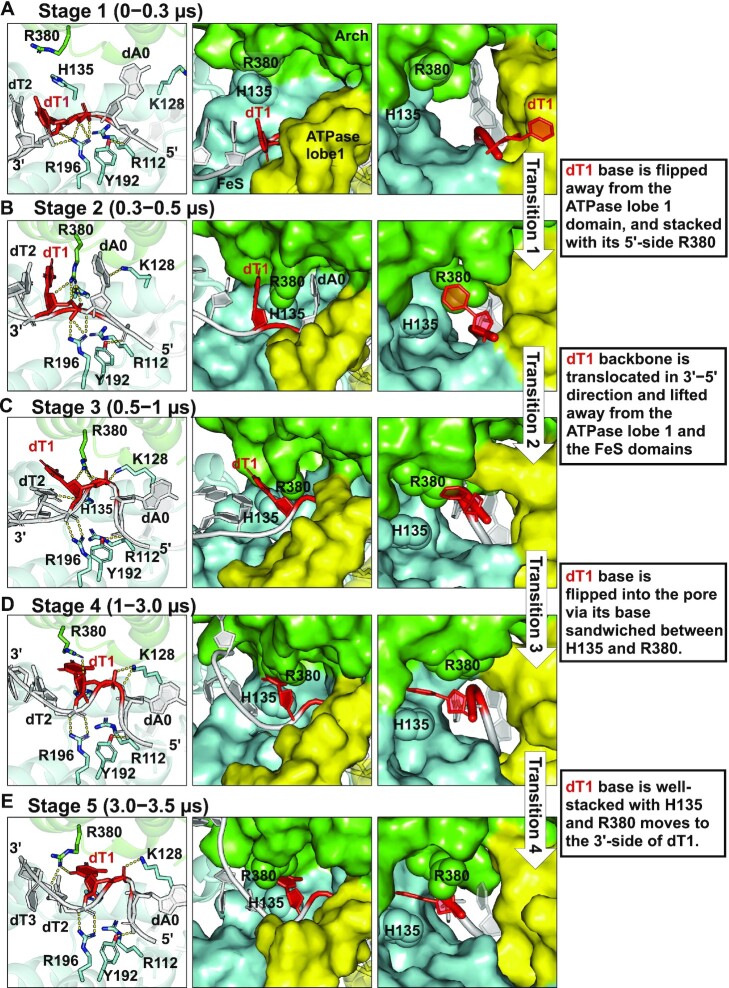
Unmodified nucleotides near the pore are translocated in the 3′→5′ direction passing through the entry pore. (**A**) *Stage 1* (during 0−0.3 μs), the nucleotide dT at position 1 (dT1) is placed outside the pore with its base pointing toward the ATPase lobe 1 domain. R196 (FeS) anchors the backbones of dT1 and dT2. Y192 (FeS) interacts with backbone of dA0. (**B**) *Stage 2* (during 0.3−0.5 μs), the side-chain of R380 (Arch) is inserted between dT1 and dA0. The positively-charged side-chain of R380 is positioned on the 5′-side of dT1 and forms cation-pi interactions with the base of dT1 (Supplementary Figure S9); thus, it aids in pulling the base of dT1 toward the Arch domain and away from the ATPase lobe 1 domain. Interactions of R196 with dT1 and Y192 with dA0 remain unchanged. (**C**) *Stage 3* (during 0.5−1 μs), R380 (Arch) and K128 (FeS), both positioned on the 5′-side of dT1, together pull the phosphate group of dT1 to outcompete its backbone interactions with R196; as a result, the backbone of dT1 is translocated toward its 5′ direction and is pulled toward the Arch domain and lifted away from the FeS and the ATPase lobe 1 domains. A bend is formed between dA0 and dT1 because the phosphate group of dA0 is still anchored stably by Y192 after the backbone of dT1 is translocated in the 3′−5′ direction. Now, R196 interacts with dT2 and the side-chain of R380 on the 5′-side of dT1 retains its cation-pi interactions with the base of dT1. (**D**) *Stage 4* (during 1−3.0 μs), the base of dT1 is flipped into the pore with its base sandwiched between H135 and R380; in this way, the base of dT1 avoids a steric clash with the side-chain of R380. The bend between dT1 and dA0 is retained. Contacts between R196 and dT2, Y192 and dA0, and K128 and the dT1 backbone remain unchanged. (**E**) *Stage 5* (during 3.0−3.5 μs), R380 has moved to the 3′-side of dT1 and interacts with the backbone of dT2 and dT3. The base of dT1 is still well-stacked with H135. The DNA bend between dT1 and dA0 is retained (also see Supplementary Figure S11). Interactions of R196 with dT2, Y192 with dA0, and K128 with the dT1 backbone remain unchanged. Note that the best representative frames from each stage are displayed, revealing the pathway of the nucleotides’ translocation in the 3′→5′ direction along the entry pore (also see Supplementary MovieS1). Left panel shows the XPD–ssDNA interactions near the entry pore; the hydrogen bonds are denoted by the yellow dashed lines. The middle panel is a view along the entry pore (surface rendering), showing the positioning of the nucleotides along the pore during the MD simulation. Right panel shows a view into the entry pore; 3′-end d(TTG) nucleotides are not shown for clarity. The structures are color-coded as in Figure 1. H135 and R380 are labeled and displayed as sphere rendering, highlighting their relative positions with respect to the undamaged dT1.

In the case of XPD-unmod in the 3.5 μs simulation, our results show that the undamaged nucleotides initially positioned outside the pore undergo a large displacement, mainly reflected in their backbone 3′→5′ translocation and the reorientation of the bases (Figure 2A). Notably, dT1 that was initially positioned right outside the pore is translocated into the entry pore, indicating that the XPD protein allows the undamaged DNA to pass through. This translocation occurs during 0–1 μs via multiple conformational transitions that take place over hundreds of nanoseconds (Figure 3, Supplementary MovieS1).

These multiple conformational transitions of dT1 are predominantly driven by a dynamic network of interactions of dT1 with surrounding XPD pore residues, including R112, K128, H135, Y158, R196 from the FeS domain as well as R380 from the Arch domain. At the initial structure, the Arch residue R380 has its side chain pointing toward the ATPase lobe 1 helix, leading to a closed state of the pore (see initial frame in Supplementary Figure S5A−B); the dT1 is positioned outside the pore with R380 on its 5′-side and its T base is oriented toward the ATPase lobe 1 helix (Model 3 in Figure 1B, Supplementary Figure S2A); the nucleotides at positions 0–2 (dA0, dT1, and dT2) near the pore are mainly contacted by the FeS residues R112, Y158, and R196 via their backbone: dT1 by R112 and R196, dT2 by Y158, and dA0 by R112 and Y192 (Model 3 in Figure 1C). At *Stage 1* (∼0−0.3 μs, Figure 3A), in the very early stage (within a few nanoseconds) of the simulation, we observed an increased distance between the Arch helix and the ATPase lobe 1 helix, reflected in the increase in the Cα(R380)−Cα(L220) distance (Supplementary Figure S5A); also, the side chain of R380 points away from the ATPase lobe 1 helix (Supplementary Figure S5B). These rearrangements result in an opened gap between the Arch and the ATPase lobe 1 domains right at the DNA entry pore (Supplementary Figure S5C). Furthermore, the contacts of R112 with the dT1 backbone and Y158 with dT2 are lost within tens of nanoseconds. At *Stage 2* (∼0.3−0.5 μs, Figure 3B), R380 (Arch) that is on the 5′-side of dT1 attracts dT1 via cation-pi interactions with the T base (Supplementary Figure S9) and hydrogen bonds with its backbone; this causes the dT1 base to reorient toward the Arch domain and to release its T base contact from the ATPase lobe 1 domain, as reflected in their abolished van der Waals interactions (Supplementary Figure S10A). At *Stage 3* (∼0.5−1 μs, Figure 3C), R380 (Arch) and K128 (FeS) together pull the phosphate backbone of dT1, which outcompetes its interactions with R196 (FeS); as a result, R196 loses its contacts with dT1 and interacts with its 3′-side nucleotide, dT2, instead. Furthermore, the ATPase lobe 1 helix is greatly tilted away from the DNA, leading to an even more opened state of the pore (Supplementary Figure S7B). Thus, these rearrangements cause the dT1 backbone to be lifted away from the FeS and the ATPase lobe 1 domain, allowing the phosphate moiety of dT1 to be pulled toward its 5′ direction and toward the Arch domain. Notably, a DNA backbone bend between dA0 and dT1 that we observed occurs because the dT1 backbone is pulled toward its 5′-direction while the dA0 backbone is still anchored stably with Y192. At *Stage 4* (∼1−3.0 μs, Figure 3D), the T base is flipped into a position where it is sandwiched between H135 (FeS) and R380 (Arch) to enter the pore; in this way, the T base avoids a steric clash with the side chain of R380. The DNA backbone contacts, including dT2 with R196, dA0 with Y192, and dT1 with K128, remain unchanged and the DNA bend between dA0 and dT1 retains. At *Stage 5* (∼3.0−3.5 μs, Figure 3E), the side chain of R380 has moved to the 3′-side of dT1 and interacts with the backbone of dT2 and dT3; this ensures that the dT1 is completely within the pore while retaining its base stacking interactions with H135 (FeS). Other key residues stabilizing this local conformation are R196 with dT2, K128 with the dT1 backbone, and Y192 with the dA0 backbone. We note that the DNA bend between dT1 and dA0 is still retained; it cannot be eliminated until dA0 releases its contact with Y192 or more 5′-nucleotides release their contact with the XPD protein. Overall, our simulation showed that the dT1 translocation along the entry pore was mainly achieved before ∼1 μs (Figure 3A–C) and its position remains then stable due to its T base stacking interactions with H135 (Figure 3D−E). However, we observed side-chain movement of R380 throughout the simulation: this side chain moved from the position on the 5′-side of dT1 (*Stages 2 and 3*, during 0.3−1 μs, Figure 3B−C) to its stacked position with the dT1 base (*Stage 4*, during 1–3 μs, Figure 3D), and then to the position on the 3′-side of the dT1 where it interacts with the backbone of dT2 and dT3 (*Stage 5*, during 3–3.5 μs, Figure 3E); this movement suggests a possible role for R380 in supporting the 5′→ 3′ translocation of XPD with undamaged DNA.

A DNA bend between the dT1 and dA0 occurred within the pore during the dT1 backbone translocation (*Stage 3*, Figure 3C), which is retained throughout the simulation (*Stages 3 to 5*, Figure 3C–E). We have superimposed the structures from *Stage 1* and *Stage 5*, as shown in Supplementary Figure S11. We did not find that displacement of the Arch domain, *i.e*. lifting away from the DNA, contributes to the extra space needed for this DNA bend. However, we did observe a movement of the dA0 base that is flipped away from its initial position within the pore (Supplementary Figure S11A); this creates the space for the bend, as well as for the side chain of K128 that points to the backbone of dT1. We also observed displacement of the ATPase lobe 1 helix away from the DNA, enlarging the pore width to allow the DNA to enter (Supplementary Figure S11B). Furthermore, this DNA bend has a relatively close phosphodiester distance of ∼8 Å across the loop (zoom-in view in Supplementary Figure S11A); three positively-charged residues K128, R196 and R380 bind to the backbone of the DNA bend to balance out the negative repulsion caused by the close phosphodiester distance in the looped DNA, explaining the persistence of the bend throughout the simulation. This energy-balancing strategy in our case is similar to that of the experimentally observed Mre11–ssDNA complex observed by Williams *et al.* (67).

### In the lesion-containing XPDs, the 6−4PP lesion is immobilized and blocked from backbone 3′→5′ translocation via its extensive interactions with the XPD pore

In contrast to the case of XPD-unmod where the unmodified DNA undergoes 1nt backbone translocation in the 3′→5′ direction through the entry pore (Figures 2A and 3), this noticeable backbone translocation is absent for the 6−4PP lesion in both lesion-containing XPDs (Figure 2B and C) when comparing the structure from the equilibrated ensemble with its initial structure for each XPD case; this is reflected in the small values of P atom RMSDs of the lesion, which are much less than the B-DNA phosphate-phosphate distance of ∼7 Å. The hindered translocation of the lesion is due to its extensive interactions with the XPD pore, mainly involving the FeS residues that interact stably with the backbone of the lesion as well as the ATPase lobe 1 residues that interact tightly with the modified bases (Supplementary Table S1 and Figure S10). Notably, the bases of the 6−4PP lesion are oriented toward the ATPase lobe 1 domain (Figure 2B and C, Supplementary Figure S10B and S10C), while in XPD-unmod, the T base is pointed toward the interface between the Arch and the FeS domains and flipped away from the ATPase lobe 1 (Figure 2A, Supplementary Figure S10A), indicating that the ATPase lobe 1 domain is responsible for verifying the presence of the modified bases.

**Figure 4.**
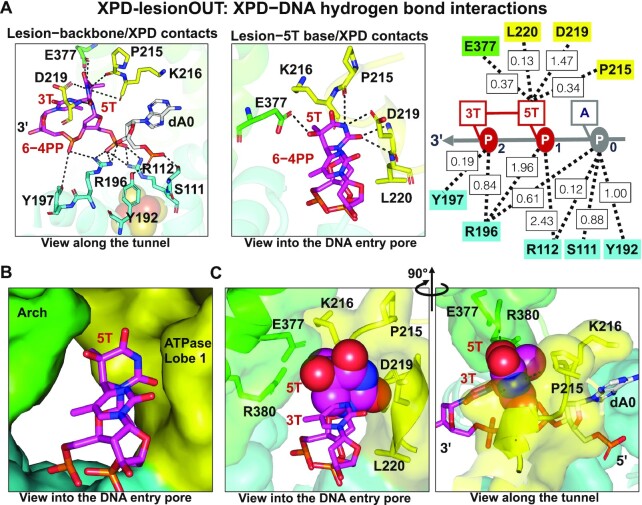
Interactions of the 6−4PP lesion with surrounding XPD residues in XPD-lesionOUT. (**A**) The hydrogen bonding interactions of XPD residues with the backbone and bases of the lesion are shown in the left and middle panels, respectively. Right panel, schematic representation of hydrogen bonds (dashed lines) of XPD residues with the 6−4PP lesion; the hydrogen bond numbers of each residue with nucleotides are also displayed. Total hydrogen bond numbers of each XPD residue−nucleotide pair are the summation of the values of all the hydrogen bond donor-acceptor pairs within each XPD residue−nucleotide pair, as listed in Supplementary Table S2. For details see hydrogen bonding analysis in Supplementary Structural Analyses. The backbone of the 6−4PP lesion is anchored stably by the FeS residues R196, Y192 and R112, with hydrogen bond number (total HBs) ∼5.42; the bases of the lesion interact with the ATPase lobe 1 residues P215, D219, and L220, with total HBs ∼1.95 and the Arch residue E377 (Arch) with total HBs ∼0.30. (**B**) The 5T of the lesion is clamped narrowly between the Arch (E377and R380) and the ATPase lobe 1 (residues 215–221) domains. (**C**) The N3 and O2 atoms of the 5T base (rendered in spheres) are captured by a small pocket formed by the ATPase lobe 1 helix. Furthermore, the loop with residues P215 and K216 of the ATPase lobe 1 domain is inserted between dA0 and the 5T base of the lesion, aiding in blocking the 5T base of the lesion from entering the pore (also see Supplementary MovieS2).

The strong grip of the XPD pore on the lesion is manifested by an extensive network of interactions that stabilize the 6−4PP lesion outside or within the pore. For XPD-lesionOUT where the lesion is initially positioned outside the pore with it bases toward the Arch and FeS domains, the phosphate moieties of the 6−4PP are anchored stably by FeS residues R112 and R196 (Figure 4A); however, the modified bases reorient as they transit from state 1 (during 0–1 μs) to state 2 (1–2 μs), and to the equilibrated state (2–3.5 μs) (Figure 2B and Supplementary MovieS5). In the equilibrated state, the modified 5T base of the 6-4PP lesion is clamped tightly between the Arch and the ATPase lobe 1 domains mainly through hydrogen bonding and van der Waals interactions (Figure 4 and Supplementary Figure S10B). Notably, we found that the lobe 1 residues P215, D219 and L220 form hydrogen bonds with the N3 and O2 atoms of the 5T base of the 6−4PP, further driving the 5T base of the lesion to reorient toward the ATPase lobe 1 domain; thus, the N3 and O2 atoms of the 5T base are captured by a small pocket formed by the ATPase lobe 1 helix with residues 215−221 (PKIADLV), comprised of several hydrophobic residues. Furthermore, the lobe 1 residues P215 and K216 are inserted between dA0 and the 5T of the lesion, aiding in blocking this 5T base from entering the pore (Figure 4C and Supplementary Movie S2).

**Figure 5.**
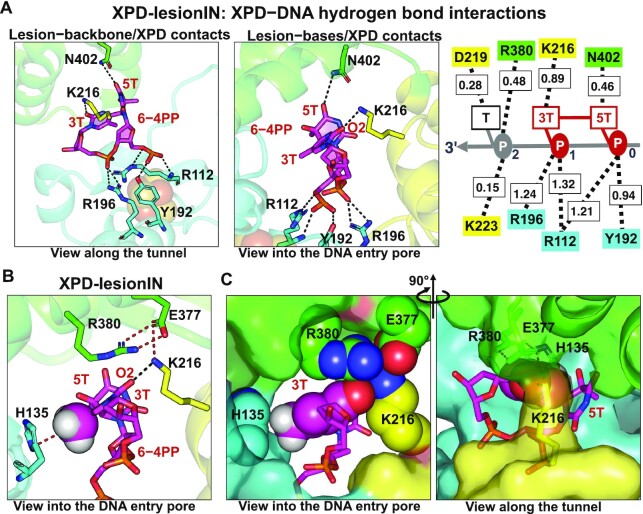
Interactions of the 6−4PP lesion with surrounding XPD residues in XPD-lesionIN. (**A**) The hydrogen bonding interactions of the XPD residues with the backbone and bases of the lesion are shown in the left and middle panels, respectively. Right panel, schematic representation of hydrogen bond interactions of XPD with the nucleotides near the pore. The backbone of the 6−4PP lesion is anchored by the FeS residues R196, Y192 and R112, with total HBs ∼5.02. The bases of the lesion contact K216 (ATPase lobe 1) and N402 (Arch) with total HBs ∼1.37. The O2 atom of the 3T base hydrogen bonds stably with K216 (ATPase lobe 1). (**B**) Other non-H-bond interactions of XPD with the 3T base of the lesion contribute to the stabilization of the 6−4PP lesion at the pore. Left panel reveals that K216 (ATPase lobe 1) which hydrogen bonds with the O2 atom of 3T forms a network of interactions with E377 and R380; furthermore, the methyl group of the 3T base of the lesion interacts with the aromatic residue H135 via methyl−π interactions. (**C**) The 3T base (rendered in spheres) of the lesion is confined between all three XPD domains: FeS, Arch and ATPase lobe 1; E377 and R380 (Arch), K216 (ATPase lobe 1), and H135 (FeS) are in close contact with the 3T base of the lesion (also see Supplementary MovieS3).

In the case of XPD-lesionIN where the bases of the 6−4PP lesion are initially positioned within the pore, our simulation showed that the backbone of the lesion is stably anchored by the FeS residues R112, Y192 and R196 (Figure 5A) and the lesion reaches a stable conformation after ∼0.6 μs (Figure 2C). In the equilibrium state, the modified bases are stabilized by all three XPD domains, mainly through hydrogen bonding and van der Waals interactions (Figure 5, Supplementary Figure S10C). Notably, the 3T base of the 6-4PP lesion is tightly-squeezed by the Arch, the FeS, and the ATPase lobe 1 domains. Residues K216, E377, R380, and H135 form a network of interactions with 3T of the lesion (Figure 5C, Supplementary MovieS3): the aromatic residue H135 (FeS) displays *Me*−π interactions with the methyl group of the 3T base (Figure 5B); K216 (ATPase lobe 1) that forms stable hydrogen bonds with the O2 atom of 3T base interacts stably with E377 and R380, leading to a closed gap between the Arch and the ATPase lobe 1 domains (Figure 5C, Supplementary Figure S5E); E377 and R380 (Arch) form van der Waals interactions with the 3T (Figure 5C, Supplementary Figure S10C). Thus, these interactions collectively shrink the pore size (Supplementary Figure S7B) to prevent this bulky lesion from further translocating in the 3′→5′ direction. We note that this extensive network of interactions that immobilize the 6−4PP lesion is not affected by the presence of the partially disordered FeS helix (residues 128–138) (Supplementary Figure S4), as observed in the simulation of XPD-lesionIN (Supplementary MovieS4). Biologically, we speculate that the flexibility induced by the disordered FeS helical residues might contribute to impeding translocation in lesion verification, as the interactions of these residues with the DNA may be required for the translocation process.

### Varying conformations of the extended DNA outside the pore are sampled that suggest the presence of lesion sensors in the vicinity of the entry pore

The extended single-stranded DNA protruding outside the pore, with no coordinates deposited in the cryo-EM structure, is highly flexible with varying conformations that are accessible by the MD simulations of our standard B-DNA initial model. To further explore the conformations of the extended DNA in the vicinity of the entry pore, we have carried out additional 8 independent MD simulations of ∼2 μs each for lesion-free XPD. In these additional eight simulations, after 1.5 μs, both XPD and undamaged DNA near the XPD pore reach a stable conformation; hence, we utilized the simulations between 1.5 and 2 μs to capture the properties of the equilibrated states. During the equilibrated states, the XPD retains a correctly folded structure with RMSDs of ∼1.4–1.7 Å from its initial fold (Supplementary Figure S12A), while dT1 exhibits varying deviations from its initial conformation, with heavy atom RMSD values ranging from ∼3 to 10 Å; the deviation of dT1 stems mostly from its base reorientation as its phosphate backbone retains its position within the XPD pore, which is reflected in the P atom RMSD of 0.9–3.6 Å (Supplementary Figure S12B). Combining these 8 simulations with the ∼3.5 μs MD simulation of XPD-unmod, there are a total of nine simulations for the lesion-free XPD; notably, these 9 simulations, taking the last 500 ns for each case, showed that the undamaged dT1 initially positioned outside the pore is flipped into the unoccupied space within the pore via base translocation (revealed in two of nine simulations, *i.e*. 22% of the population) or blocked from translocating via entrapment of its base (six of nine simulations, *i.e*. 67% of the population) (Supplementary Figure S13), and with 11% (one of nine simulations) of the population undergoing the dT1 backbone translocation into the entry, observed in our ∼3.5 μs MD simulation of XPD-unmod (Figure 3).

#### The blockage of dT1 suggests the presence of lesion sensors in the vicinity of the entry pore

The blockage of dT1 revealed in these simulations is mainly due to its T base being trapped by two base sensor regions in the vicinity of the entry pore (Supplementary Figure S14). One is the FeS pocket (Supplementary Figure S14C), formed by several hydrophobic residues (H135, A139, S140, Y141, Y158, F161, Y192, F193 and H384), which lies in a region that had been identified as a lesion sensor (47,52,60,68). The second is the ATPase lobe 1 helix with residues 215−221 (PKIADLV), which lies in the region right at the edge of the pore (Supplementary Figure S14B). We noticed that this ATPase lobe 1 helix captures the analogous unmodified dT1 base and the modified 5T base of the 6−4PP lesion in the XPD-lesionOUT case in a very similar way: the O2 and N3 atoms of the base are captured by a small pocket of the ATPase lobe 1 helix. However, due to the bulk and rigidity of the covalently crosslinked 6−4PP dimer with perpendicular bases, the lesion is completely obstructed from translocating, while the single native base that will translocate would only be temporarily impeded. The ATPase lobe 1 helix is right at the pore and the FeS pocket is further away from it, suggesting that a base can still be captured by the ATPase lobe 1 if it escapes from the FeS pocket. Overall, these results suggest that the lesion sensors may serve to initially recognize modified bases, while the lower population manifesting translocation (22% showing base translocation and 11% showing base and backbone translocation) reflects the existence of a modest kinetic barrier imposed by the sensors that must be overcome.

## DISCUSSION

XPD is a main sensor for verifying the presence of bulky DNA lesions in NER. An early study of the yeast XPD (Rad3) homologue by Naegeli and co-workers showed that the helicase activity of XPD is suppressed at the damaged sites (63). This has been further supported by other studies of XPD (40,43,52) and explained by the lesion-induced steric blocking of translocation; the lesion cannot pass through a narrow tunnel, which has just enough space to accommodate native DNA (53,54,56). A single-molecule imaging assay of the microscopic motion of the XPD demonstrated that the XPD pore undergoes a thermally driven open-close transition in the presence of undamaged DNA and the closed conformation of the pore is somewhat enhanced in the presence of the CPD lesion (61). Structural studies have characterized XPD interactions with the ssDNA (39,57) identifying the entire DNA binding path, the entry pore and the adjacent lesion recognition pocket (1,54). However, XPD bound with lesion-containing ssDNA that contacts the entire binding path within the XPD, and dynamic information for both damaged and undamaged DNA that contains the DNA extension outside the entry pore remain unexplored. Molecular and dynamic delineations of how XPD translocates on the undamaged DNA and blocks DNA lesions near the entry pore presented here offer new insights on the key process of lesion verification. In the present study, we have carried out ∼3.5 μs MD simulations of the XPD bound to the lesion-containing ssDNA and of an XPD complex with lesion-free ssDNA as a control (Figure 1). Additional eight ∼2 μs MD simulations for the lesion-free XPD were further performed to explore the conformations of the flexible DNA extension for the undamaged DNA (Supplementary Figures S12-S14). Furthermore, we have carried out an additional ∼2 μs MD simulation for the original cryo-EM structure with PDB ID 64RO4 (39) of XPD–ssDNA *without* the DNA extension to its 3′-end (XPD-unmod-noExtendedDNA) as a control to investigate the impact of the flexible DNA extension on the structure and dynamics of the XPD pore residues (Supplementary Figure S6).

Our ∼3.5 μs all-atom MD simulations of the human XPD in complex with ssDNA containing a 6−4PP photolesion (64) outside and within the entry pore, together with a similar unmodified DNA simulation with 3.5 μs production run for comparison, has revealed a one nucleotide backbone translocation for the unmodified case while displaying stalling in the presence of 6−4PP lesion (Figure 2). In particular, a striking finding is that when scanning the undamaged DNA near the pore, the XPD allows the unmodified nucleotides to be translocated in the 3′−5′ direction, passing through the flexible and enlarged entry pore (Figure 2A). This movement is initiated by the *opposite* motion of the Arch domain with respect to the ATPase lobe 1 domain and is subsequently driven by weakened and dynamic XPD-DNA interactions, including detachment of the native base from the ATPase lobe 1 and lifting of the sugar phosphate moiety away from the sensor pocket in the FeS domain. However, this translocation is absent for the 6−4PP lesion near the entry pore (Figure 2B and C); especially for the lesion within the pore, the XPD immediately narrows the pore to grip the lesion tightly (Figure 5). Notably, the ATPase lobe 1 helix holds the modified bases tightly and the FeS residues R112 and R196 anchor the lesion's sugar-phosphate backbone stably in both lesion-containing XPDs (Figures 4 and 5), while these stable interactions are completely absent in the lesion-free XPD (Supplementary Table S1 and Figure S10). These results highlight the ability of the XPD to discriminate the damaged DNA from the undamaged via key residues responsible for stalling the XPD.

### With undamaged DNA near the pore, the ATPase lobe 1 domain releases its hold on the DNA and the Arch domain, resulting in a flexible, open pore that permits the DNA to enter

Our 3.5 μs simulation of the XPD complex with the undamaged ssDNA revealed one nucleotide 3′→5′ translocation of the 3′-end ssDNA near the entry pore, showing XPD allowing unmodified DNA to enter the pore (Figure 2A). This phenomenon is predominantly driven by the dynamic network of interactions between the undamaged DNA and the surrounding XPD pore residues R112, R196, K128 and H135 (FeS), and R380 (Arch) (Figure 3, Supplementary MovieS1). To permit a single nucleotide to enter the pore, *first* the native base must be released from the ATPase lobe 1 domain via cation-pi interactions with the Arch residue R380 (Transition 1 in Figure 3). *Second*, its backbone needs to be released from the FeS residues R196 and R112 within the lesion-sensor pocket in close vicinity to the pore via the aid of R380 and K128, to permit a 3′→5′ backbone translocation (Transition 2 in Figure 3). *Third*, a flexible base is required which can orient freely to avoid steric clashes when flipping into the pore via its base sandwiched between H135 and R380 (Transition 3 in Figure 3). Notably, when comparing *Stage 1* and *Stage 5* in Figure 3, R196 (FeS) switches its contacts from dT1 to its 3′-dT2, and R380 (Arch) has moved from the 5′-side of dT1 to its 3′-side and interacts with the backbone of dT2 and dT3; these alterations manifest the XPD translocation in the 5′−3′ direction (1,57). Overall, these findings support the mechanism of the 5′−3′ directionality of XPD translocation on ssDNA, proposed by Cheng *et al.*(57); they reported two crystal structures of the *Escherichia coli* homolog DinG bound to ssDNA in the presence and absence of an ATP analogue (ADP·BeF_3_): a binary complex of DinG-DNA and a ternary complex of DinG-DNA-ADP·BeF_3_. By comparing the increased interactions of the HD1 with 3′–ssDNA in DinG-DNA-ADP·BeF_3_ and HD2 with 5′–ssDNA in DinG-DNA, they proposed a model suggesting that when ATP is bound, the HD2 domain slides along the ssDNA toward its 3′-end, while HD1 retains a tight grip on the 3′-end of the ssDNA. After the ATP hydrolysis, HD1 releases its hold on the ssDNA to allow one nucleotide to translocate toward the 5′−end, as expected for 5′−3′ translocation of XPD, while the HD2 grips the 5′−end of the DNA tightly. Therefore, based on their observations, we speculate that with ATP hydrolysis, HD1 releases its hold on the 3′–ssDNA, facilitating the translocation of one nucleotide toward the 5′-end. We further surmise that when the XPD encounters a bulky lesion near the entry pore, the XPD grips the lesion so tightly that it is unlikely to release it even after ATP hydrolysis, and therefore, translocation would be inhibited at the lesion site. Lesion size, shape and chemical features would be expected to modulate the extent that translocation is inhibited. Together with our eight additional MD simulations for the lesion-free XPD (Supplementary Figures S13 and S14), we propose that the backbone translocation of dT1 into the pore, while relatively low in population (11%), offers biologically important and novel structural and dynamic insights on how the non-equilibrium helicase translocation process may take place during the ATP binding/hydrolysis cycle (65,66).

Another interesting question concerns how well conserved are key residues that we observe interacting with DNA. According to the sequence alignment of five different XPD proteins from *T. acidophilum*, human, mouse, *Arabidopsis thaliana* and *Saccharomyces cerevisiae* reported by Kisker *et al.* (56), most key residues involving the DNA translocation or lesion verification revealed in our MD simulations are conserved residues, except K128, H135, and R380. Our simulations have shown that for the undamaged DNA, R380 and K128 are responsible for the DNA backbone translocation that causes a DNA bend; R380 and H135 are involved in the base flipping into the entry. Therefore, we speculate that replacing any of these three non-conserved residues may affect function, perhaps altering the rate of helicase activity. Also, an R380A mutant may diminish the interactions required for dT1 backbone translocation; however, with R380A, the dT1 base might flip into the pore more efficiently due to the reduced steric effect of the Ala residue.

At a very early stage of our 3.5 μs MD simulation for the lesion-free XPD, the Arch helix containing R380 exhibits opposite movement with respect to the ATPase lobe 1 helix (Supplementary Figure S5A), and the side chain of R380 points away from the ATPase lobe 1 helix (Supplementary Figure S5B); these rearrangements result in a noticeable gap between the Arch and ATPase lobe 1 domains right at edge of the entry pore (Supplementary Figure S5C), leading to an opened state of the pore that initiates the changes in the interactions between these two domains as well as between the XPD and the DNA. This gap persists throughout the simulation and is dynamic (Supplementary Figure S5A–B). As a result, both Arch domain and the DNA become more mobile; at ∼0.5 μs, the Arch domain exhibits dynamic movement with respect to the FeS domain (Supplementary Figure S7D) and the DNA is translocated through the pore (Supplementary Figure S7C), concomitant with the great pore enlargement (Supplementary Figure S7B). These findings are consistent with experimental studies (46,61,69,70), suggesting a role for the Arch domain in regulating the size and dynamics of the pore and in the translocation cycle. In one experimental study by Ghoneim and Spies (61), their single molecule investigations showed that the Arch domain undergoes thermally driven open-close transitions when undamaged DNA binds. In the Naismith laboratory (46), it was demonstrated that locking the Arch and the FeS domains in the closed state inactivates the XPD helicase activity, suggesting that the Arch has to disengage from the FeS domain, leading to a transient opened pore that permits the DNA to enter.

We noted that the Arch domain has an extremely mobile region (so called ‘plug’) that is not visible in the cryo-EM structure of XPD bound to ssDNA with PDB ID 6RO4 (39); thus, we have excluded this Arch plug (details see Materials and Methods) in our simulations. A structural study (55) showed that when MAT1 (ménage a trois 1) binds to the Arch domain, the Arch plug occupies the DNA entry pore and blocks DNA binding. On the other hand, without MAT1, the Arch plug is displaced and mobile, allowing XPD to bind to ssDNA. Kisker and colleagues reported a crystal structure of the human XPD, in which the Arch domain is in complex with MAT1 (69); this structure revealed that the XPD is negatively regulated by the presence of MAT1, which binds directly with the Arch domain to reduce the DNA binding capacity of XPD; after release from MAT1, it was suggested that the positively charged area of the Arch domain may engage with the non-translocating DNA strand. Therefore, in the absence of MAT1, we speculate that the Arch plug may engage with the non-translocating strand, which would not directly affect the translocated strand. Mutagenesis data also identified residues of the Arch domain functionally essential for XPD helicase activity (69,70), suggesting that the Arch domain dynamics that switches between the open and closed states aids the XPD helicase activity and strand separation. Taken together with other studies, our MD results indicate that the XPD protein around the DNA entry pore is mobile, particularly the motion of the Arch domain with respect to the FeS domain, to allow passage of the translocated strand. It is noteworthy that the local motions of the XPD pore observed here, such as a closed and opened gap, pore size variation and the relative motion of the Arch domain with respect to the FeS domain, are at a completely different scale than the community network of motions stemming from the interactions occurring in the huge multi- protein system of the human transcription preinitiation complex (PIC) investigated by Yan *et al.* (71), and the latter relates to transcription initiation where XPD functions very differently than in NER (39,45–47).

### When XPD encounters the 6−4PP lesion near the pore entry, the ATPase lobe 1 domain restrains the lesion bases for damage verification

In contrast to the case of the lesion-free XPD where the unmodified nucleotides undergo a 3′→5′ backbone translocation near the entry pore (Figures 2A, 3, and Supplementary MovieS1), our MD simulations of the lesion-containing XPDs show that this translocation is not observed for the bulky 6−4PP lesion-containing DNA (Figure 2B–C); this is in line with reports from several groups indicating that when encountering a bulky lesion on the translocated strand, the helicase activities of yeast and archaeal XPD homologs as well as human XPD with intact TFIIH are inhibited at the damaged site (40,41,43,52,63). We found that the backbone of the 6−4PP lesion is anchored stably by R196 and R112 of the FeS domain, while for undamaged dT1, these interactions are abolished (Supplementary Table S1), explaining why a 3′→5′ backbone translocation is not allowed for the lesion. Moreover, the bases of the 6−4PP lesion are restrained by the ATPase lobe 1 domain mainly via van der Waals and hydrogen bonding interactions (Figures 4 and 5, Supplementary Table S1 and Figure S10), while the ATPase lobe 1 domain releases its hold on the native base (Figure 3B–E, Supplementary Figure S10A), suggesting that the ATPase lobe 1 domain is responsible for the verification of the modified bases of this bulky lesion; the ATPase lobe 1 helix as a lesion sensor is further supported by additional simulations for the highly-flexible extended DNA (Supplementary Figures S13B and S14B). These simulations observed two lesion recognition sensors near the entry pore (Supplementary Figures S13 and S14); the purpose of these sensors for the unmodified DNA may be to regulate the translocation rate (72), and they likely play an important role in the XPD function of damage verification in NER (47,60) and helicase activity (51,53,62).

Our molecular and dynamic views of lesion verification and how it contrasts with DNA translocation when the DNA is undamaged are consistent with experimental studies (27,46,61,69,70,73). Our results elucidate how the presence of the 6−4PP lesion affects the dynamics and size of the entry pore. We found that the backbone of the 6−4PP lesion is anchored stably by the FeS domain, and its bases are clamped tightly between the Arch and the ATPase lobe 1 domains; these interactions lock indirectly the Arch to the FeS domain and greatly reduce the mobility of the Arch with respect to the FeS domain, as reflected in their stable RMSDs (Supplementary Figure S7D). As a result, these restrained domains with reduced dynamics favor a closed and less mobile pore (Supplementary Figure S5), facilitating the stalling of the XPD (Figures 4 and 5) (46). Ghoneim and Spies (61) reported that with the presence of the less bulky CPD damage, the Arch domain undergoes a thermally driven open-close transition, which slightly favors the closed state. Thus, the important role of Arch domain dynamics in XPD helicase translocation or blockage, which aids in the recruitment of XPF and XPG to coordinate successful damage removal, is highlighted (27,69,70).

In conclusion, the 5′−3′ XPD helicase acts as a main sensor for verifying the presence of a bulky lesion to avoid erroneous dual incisions on damage-free sites in NER. We have generated experimentally-based molecular models of human XPD bound to undamaged and damaged ssDNA containing the 6−4PP UV photolesion, situated at and near the XPD pore entrance. Using all-atom molecular dynamics simulations, we demonstrate how XPD responds to the presence of the damaged DNA in contrast to its handling of undamaged DNA and delineate the atomistic and dynamic mechanism utilized to discriminate damaged from undamaged nucleotides. We found that, when encountering undamaged DNA near the XPD pore, the ATPase lobe 1 domain releases its grip on the DNA and also weaken its interactions with the Arch domain; thus, the disengaged Arch domain becomes mobile, resulting in a flexible pore that is greatly enlarged when the DNA undergoes a 3′−5′ translocation passing through the pore; this is consistent with the mechanism of 5′−3′ directionality for XPD translocation on ssDNA and further highlights the roles of ATPase lobe 1 and the dynamics of the Arch in the mechanism of translocation. However, when a bulky 6−4PP lesion is near the entry, XPD immediately immobilizes it, mainly via an extensive network of interactions with the modified thymine bases. Notably, the ATPase lobe 1 domain grips the lesion bases tightly and the Arch domain is locked to the ATPase lobe 1 and the FeS domains. As a result, the pore narrows and becomes less flexible, stalling the XPD and thereby verifying the presence of the lesion. Our model provides one possible view of XPD allowing undamaged DNA to pass through its pore while inhibiting translocation of damaged DNA. Future investigation is needed to further explore the fascinating molecular and dynamic mechanisms underlying the XPD helicase's functions in translocation and lesion verification. In addition, our MD study also elucidates the role of lesion sensors that had been identified in the vicinity of the pore (47,52,60,68). We suggest that the purpose of these sensors for the unmodified DNA is to regulate the translocation rate (72) and that they present a modest kinetic barrier to translocation in this case. Thus, we obtain for the first time, dynamic molecular characterizations of the XPD helicase activity with unmodified DNA and its inhibition by the lesion; we thereby delineate the atomistic functioning of XPD in nucleotide excision repair, and also show how it is consistent with experimental findings. Future work is needed to atomistically elucidate how different lesion structures impact the verification mechanism and provide insights on the adverse molecular impact of XPD mutations associated with human diseases.

## DATA AVAILABILITY

The 3.5 μs MD simulation trajectories for XPD-unmod, and XPD-lesionOUT, and XPD-lesionIN are deposited on GitHub (https://github.com/broydelab). The trajectory files are presented in binary NETCDF format, a corresponding topology PRMTOP file and PDB file with the coordinates of the initial structure for each XPD–ssDNA system are also provided. Please note that water molecules and ions are not included in the trajectory files.

## Supplementary Material

gkac496_Supplemental_Files
