# Supplementary material for: Mechanism of lesion verification by the human XPD helicase in nucleotide excision repair
Source: Nucleic Acids Res. 2022 Jun 17;50(12):6837–53. doi: 10.1093/nar/gkac496 (PMC9262607; doi:10.1093/nar/gkac496)
Supplement: gkac496_Supplemental_Files [file gkac496_supplemental_files.zip › SI-NAR-galley.pdf]

Supplementary Data

Mechanism of lesion verification by the human XPD helicase in nucleotide excision repair

Iwen Fu[1], Hong Mu[1], Nicholas E. Geacintov[2], and Suse Broyde[1]

[1]Department of Biology and [2]Department of Chemistry, New York University, 100 Washington Square East, New York, NY, 10003, United States

Table of Contents

Supplementary Methods 2

Force field 2

Molecular dynamics simulations 2

Supplementary Structural Analyses 3

Clustering analysis 3

Analysis of the pore width and the mobility of the Arch domain 4

Analysis of hydrogen bonding between XPD and DNA 4

Analysis of the van der Waals interaction energy 5

Supplementary Tables 6

Supplementary Figures 8

Supplementary Movies 30

Supplementary References 31

Supplementary Methods

Force field

We utilized the ff14SB [1] force field and the previously published parameters for the 6−4PP lesion [2] for the MD simulations. We employed the force field parameters published by Carvalho *et al*. [3] for the FeS cluster and TIP3P [4] water for the water molecules.

Molecular dynamics simulations

All XPD-DNA complexes were explicitly solvated with TIP3P [4] water in a cubic periodic box with side length of 100 Å using the tLEAP module of the AMBER18 suite of programs [5]. Na^+ ions were added to neutralize the system and 92 $Na^+/Cl^−$ ion pairs were further added to reach the physiological salt concentration of ~ 150 mM.

All systems were subject to energy minimization, equilibration, and ~ 2.0−3.5 µs production runs of MD simulations, using AMBER18 [5] along with general-purpose graphics processing units (GPUs). The particle-mesh Ewald (PME) [6, 7] method with 9.0 Å cutoff for the non-bonded interactions was utilized in the energy minimizations and MD simulations. The SHAKE [8] algorithm and a 2 fs time step were applied for equilibration and production runs. We performed the energy minimizations in the following three stages: (1) the counterions and water molecules were minimized with 1000 steps of steepest descent followed by 1000 cycles of conjugate gradient method, with a restraint force constant of 50 kcal/(mol·Å2) on the solute molecules (DNA and protein complex); (2) a similar minimization procedure was conducted on the counterions and water molecules, with a decreased restraint force constant of 10 kcal/(mol·Å2) on the solute molecules; (3) the whole system was then energy minimized for 2000 steps of steepest descent and 2000 steps of conjugate gradient method without any restraints. We then performed energy equilibration in three rounds: (1) each system was equilibrated at constant temperature of 10 K for 30 ps with the solute molecules fixed with a restraint force constant of 50 kcal/(mol·Å2); (2) the system was then heated from 10 K to 300 K over 300 ps with the solute molecules fixed with a restraint force constant of 50 kcal/(mol·Å2) at constant volume. In the last round of equilibration, the restraint force constant on the solute was reduced through five steps: at 50 kcal/(mol·Å2) for 100 ps, at 10 kcal/(mol·Å2) for 200 ps, at 5 kcal/(mol·Å2) for 300 ps, at 1.0 kcal/(mol·Å2) for 200 ps, then at 0.1 kcal/(mol·Å2) for 300 ps, at constant pressure and 300 K. Following equilibration, production MD simulations for each

system were carried out in a constant-temperature, constant-pressure (NPT) ensemble at 300 K and constant pressure of 1 Atm for 3.5 µs. The temperature was maintained by a Langevin thermostat [9] with a 5 ps^{-1} collision frequency, and pressure was maintained by the Berendsen [10] coupling method. An extra constraint was applied between the backbone C atom of V270 and the C atom of A326 during the equilibrium and production MD runs to stabilize these two ends, which are connected by the plug of the mobile Arch domain. The trajectories were saved every 10 ps for further analysis.

Supplementary Structural Analyses

Post-processing and analysis of all simulations was performed using the CPPTRAJ [11] module of AMBER18 [5]. All mean values and standard deviations for the stable ensemble of each MD simulation were computed using the block averaging method [12, 13]. Molecular images and movies were generated with PyMOL (Schrodinger, LLC.) [14] and VMD [15].

Clustering analysis

We utilized the MD simulations between 2.0 and 3.5 µs for the ensemble analyses to capture the properties of the equilibrated states for the XPD-ssDNA cases (**XPD-lesionIN, XPD-lesionOUT and XPD-unmod**) run to ~ 3.5 µs (**Figure 2 and Supplementary Figure S3**). For the simulations run to ~ 2.0 µs (8 additional simulations of **XPD-unmod** and one **XPD-unmod-noExtendedDNA**), we utilized the simulations between 1.5 and 2.0 µs for the equilibrium ensemble analyses; the utilized ensembles all showed stability (**Supplementary Figures S12**).

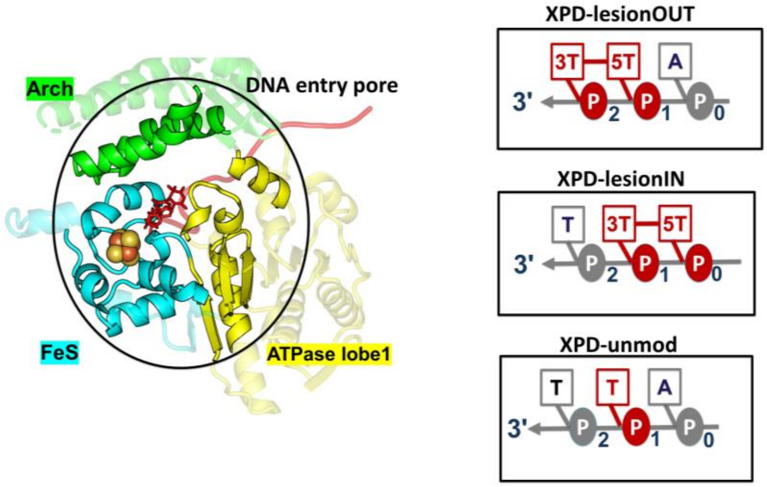

Left panel: the local region near the DNA entry pore used for clustering. Right panel: the DNA near the pore used for clustering

The most representative structures of the XPD-DNA complexes from the equilibrated states were obtained based on the local region near the entry pore (see left panel in figure left) using cluster analysis, which was performed using the average linkage hierarchical agglomerative method [16] and RMSD as the distance matrix. For the XPD protein, this local

region includes the ATPase lobe 1 residues 69−88, 104−109, 204−226, 239−247, the FeS residues 110−138, 154−164, 177−200, and the Arch residues 369−409. For the DNA, the nucleotides at positions (0, 1, 2) for each XPD-DNA complex are considered for the clustering (right panel).

Analysis of the pore width and the mobility of the Arch domain

The approximate indicator for the pore width that we monitored to evaluate how the pore width responds to undamaged versus damaged DNA, is the distance Cα(H135)−Cα(L220) (**Supplementary Figure S7B**). Note that this distance *only* involves the ATPase lobe 1 helix and the FeS helix (see figure below); hence, there is no motion information about the Arch domain in the pore width. Furthermore, the motion of this distance only represents that of two Cα atoms and hence cannot represent the total mobility of the XPD pore domains considered in the RMSDs (**Supplementary Figure S3**). In order to assess the relative motion of the Arch and FeS domains, we measured the Cα RMSDs of the Arch domain after fitting the FeS domain Cα atoms to the initial structure for each XPD-ssDNA case to obtain an estimate of this relative motion (**Supplementary Figure S7D**).

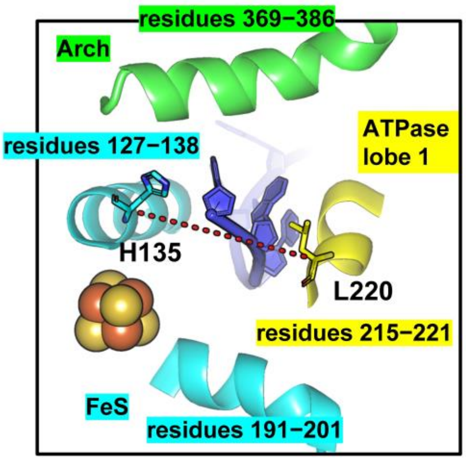

Analysis of hydrogen bonding between XPD and DNA

Hydrogen bonds of the DNA near the pore with surrounding XPD residues were counted for the equilibrium ensemble in each XPD-ssDNA case. Hydrogen bonds were counted using the criterion for each hydrogen bond pair (donor-accept pair) with hydrogen bond distance (donor-to-acceptor atom) ≤ 3.5Å and hydrogen bond angle (donor-hydrogen-acceptor) ≥ 120°. A fractional hydrogen bond between donor-acceptor is one that is present in the corresponding fraction of the population of the analyzed MD trajectories. Values for all the hydrogen bond donor-acceptor pairs between XPD residues and nucleotides, as listed in **Supplementary Table S2,** are summed to obtain the total hydrogen bond numbers of each XPD residue-nucleotide pair as listed in **Supplementary Table S1**.

Analysis of the van der Waals interaction energy

We computed the van der Waals interaction energies of the individual domains at the entry pore, including the ATPase lobe 1 residues 215−221, the Arch residues 369−409, and the FeS residues 128−138, with the base of unmodified dT1 in lesion-free XPD (**Supplementary Figure S10A**) and the modified bases of the 6−4PP lesion in the lesion-containing XPDs (**Supplementary Figure S10B-C**), using the CPPTRAJ [11] module of AMBER18 [5] for the Lennard-Jones potential. We particularly note that in the lesion-containing XPDs, the 6−4PP lesion has two bases, 5T and 3T (see figure below), which are linked by a single covalent bond and are roughly perpendicular. Therefore, we computed the van der Waals interaction energies of each individual modified base of the 6–4 PP with each XPD domain near the entry pore, as shown in **Supplementary Figure S10B-C**.

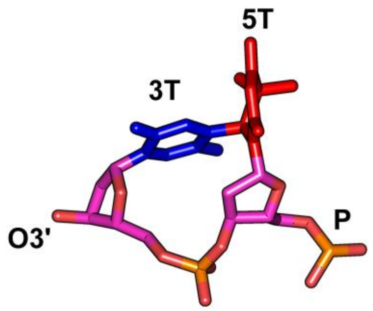

Structure of the 6−4PP lesion

Supplementary Tables

Supplementary Table S1. Hydrogen bonds between the nucleotides near the entry pore and the surrounding XPD residues in the lesion-free and lesion-containing XPDs. Only nucleotides at positions 0, 1, 2 are considered. Schematic representations involving hydrogen bonds are shown for all XPD cases (see figure below, A−C); the hydrogen bonds (black dashed-lines) of the residues to the base or to sugar-phosphate moieties of the DNA are indicated; the 6−4PP lesion and the corresponding nucleotide in the lesion-free XPD are indicated in red. Details concerning all the hydrogen bond acceptor-donor pairs within each residue-nucleotide pair are listed in **Supplementary Table S2.**

	XPD-unmod			XPD-lesionOUT		XPD-lesionIN	
**Residues**	**dT2**	**dT1**	**dA0**	**6−4PP**	**dA0**	**dT2**	**6−4PP**
**SER111**					**0.84**		
**ARG112**				**2.42**			**2.54**
**LYS113**			0.21				
**LYS128**		**0.71**	0.14				
**THR138**	0.41						
**SER140**	**0.50**						
**TYR192**			**0.99**		**1.00**		**0.94**
**ARG196**	**1.45**		0.37	**2.88**	**0.60**		**1.24**
**PRO215**				0.41			
**LYS216**							**0.89**
**ASP219**				**1.40**		0.28	
**LEU220**				0.14			
**LYS223**						0.15	
**GLU377**				0.30			
**ARG380**	**0.53**	0.27				0.48	
**ASN402**							0.46
**Total HBs**	**2.88**	**0.98**	**1.72**	**7.53**	**2.43**	**0.91**	**6.06**

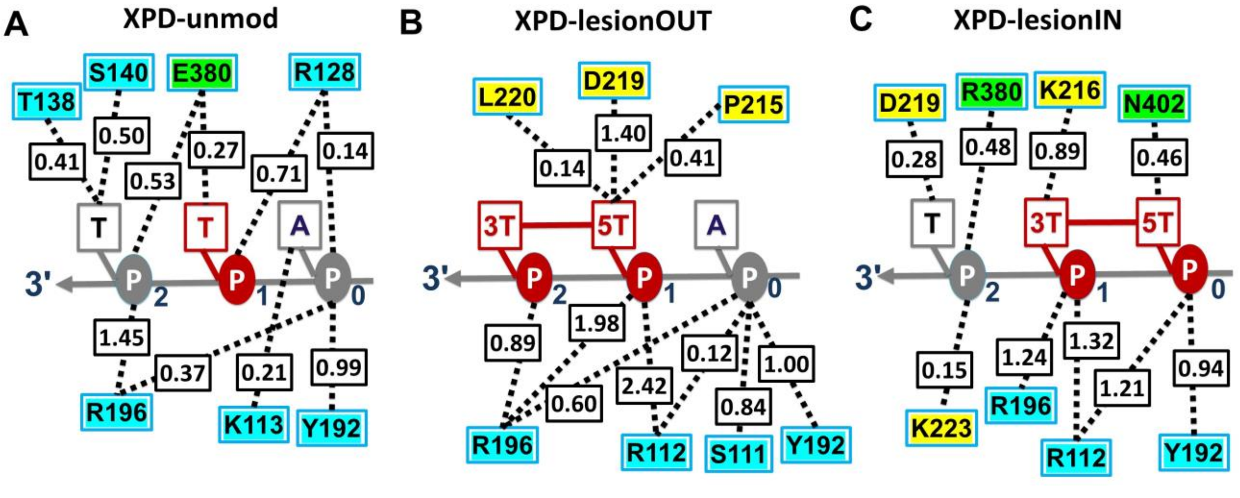

Additional note: the total HBs of the lesion with surrounding XPD residues are greater than the ones of the corresponding nucleotides in **XPD-unmod**. In **XPD-lesionOUT**, total HBs of ~ 7.5 for the lesion are greater than total HBs of ~ 3.8 for dT1and dT2; in **XPD-lesionIN**, total HBs of ~ 6 for the lesion are greater than total HBs of ~ 2.7 for dT1 and dA0.

Supplementary Table S2. Individual hydrogen bond pairs between the nucleotides and the surrounding XPD residues near the pore in the lesion-free and lesion-containing XPDs. Individual hydrogen bond pair (residue name in parentheses and atom name are given) between the DNA and residues as well as the corresponding fractional hydrogen bonds (if greater than 0.1) are listed. A fractional hydrogen bond is one that is present in the corresponding fraction of the population of the analyzed MD trajectories.

XPD-unmod

HB pair Acceptor---Donor	HB Fraction
(dT2)O3′---NH2(ARG380)	0.26
(dT2)O3′---NH1(ARG380)	0.26
(dT2)OP1---NH1(ARG196)	0.63
(dT2)OP1---NH2(ARG196)	0.58
(dT2)OP2---NH2(ARG196)	0.24
(dT2)O4---N(SER140)	0.50
(dT2)O4---OG1(THR138)	0.41
(dT1)O2---NH1(ARG380)	0.27
(dT1)OP2---NZ(LYS128)	0.49
(dT1)OP1---NZ(LYS128)	0.22
(dA0)OP1---NH1(ARG196)	0.20
(dA0)OP1---NH2(ARG196)	0.18
(dA0)OP2---OH(TYR192)	0.99
(dA0)O3′---NZ(LYS128)	0.14
(dA0)N7---NZ(LYS113)	0.21

XPD-lesionOUT

HB pair Acceptor---Donor	HB Fraction
(6-4PP)OP1---NH2(ARG196)	0.99
(6-4PP)OP1---NH1(ARG196)	0.99
(6-4PP)O5P---NH1(ARG196)	0.89
(6-4PP)OP2---NH1(ARG112)	0.95
(6-4PP)OP2---NH2(ARG112)	0.93
(6-4PP)OP1---NH1(ARG112)	0.53
(6-4PP)O2---N(LEU220)	0.14
(6-4PP)O2---N(ASP219)	0.76
(dA0)O3′---NH2(ARG196)	0.59
(dA0)OP1---OH(TYR192)	1.00
(dA0)OP2---OG(SER111)	0.84
**Donor---Acceptor**	**Fraction**
(6-4PP)O4T---OE1(GLU377)	0.16
(6-4PP)O4T---OE2(GLU377)	0.14
(6-4PP)N3---OD1(ASP219)	0.35
(6-4PP)N3---OD2(ASP219)	0.28
(6-4PP)N3---O(PRO215)	0.41

XPD-lesionIN

HB pair Acceptor---Donor	HB Fraction
(6-4PP)O4---ND2(ASN402)	0.46
(6-4PP)O2T---NZ(LYS216)	0.88
(6-4PP)O5P---NH1(ARG196)	0.73
(6-4PP)O5P---NH2(ARG196)	0.52
(6-4PP)OP1---OH(TYR192)	0.94
(6-4PP)O4P---NH1(ARG112)	0.99
(6-4PP)OP2---N(ARG112)	0.95
(6-4PP)O4P---NE(ARG112)	0.33
(6-4PP)O5′---NE(ARG112)	0.26
(dT2)OP2---NH2(ARG380)	0.30
(dT2)OP2---NE(ARG380)	0.18
(dT2)O3′---NZ(LYS223)	0.15
**Donor---Acceptor**	**Fraction**
(dT2)N3---OD2(ASP219)	0.15
(dT2)N3---OD1(ASP219)	0.13

Supplementary Figures

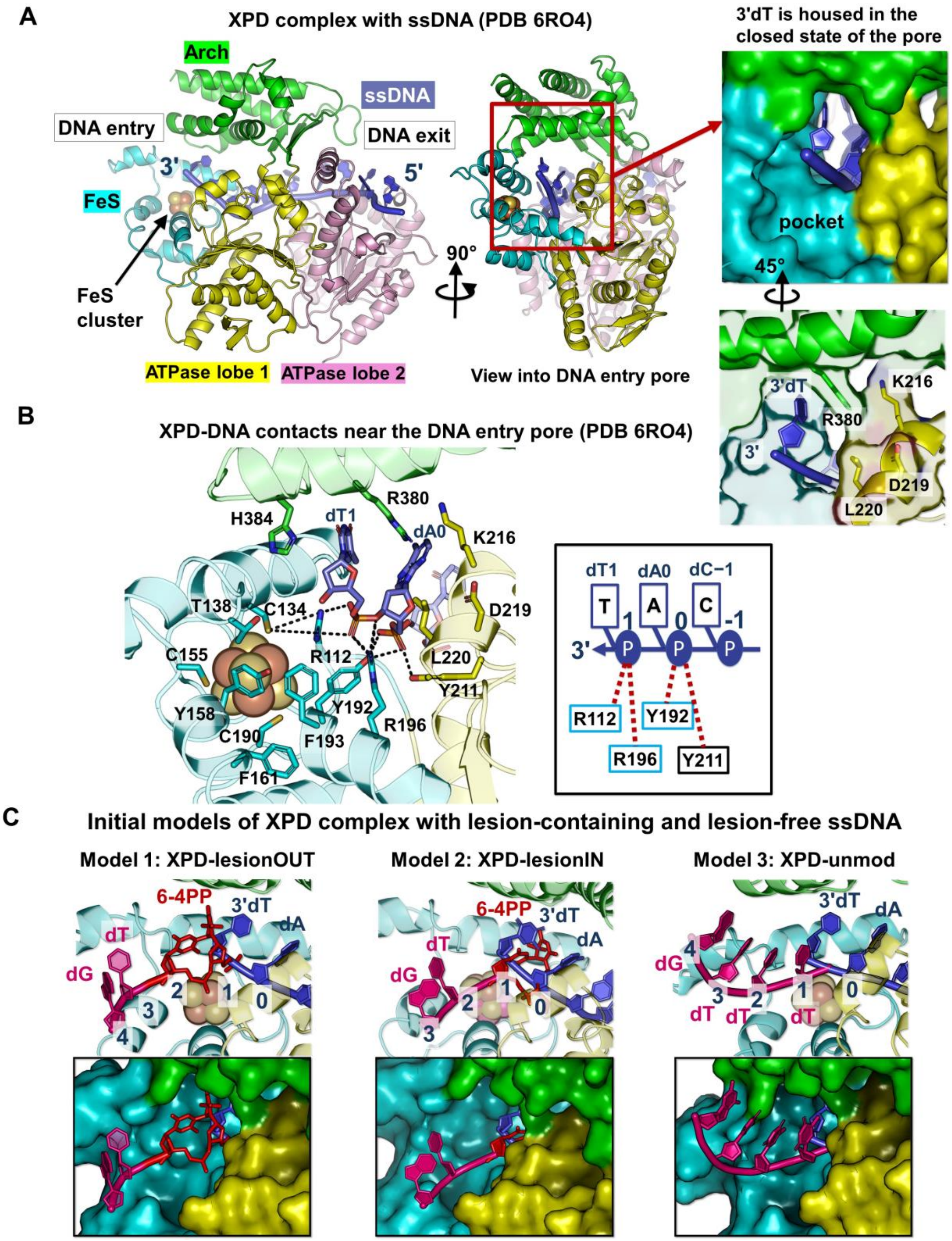

Supplementary Figure S1. Overall view of XPD bound to 11nt-ssDNA in human core TFIIH complex (PBD ID 6RO4 [17]) and the initial models of XPD bound to lesion-containing and lesion-free ssDNA used for the MD simulations.

(A) Overall cryo-EM structure of XPD complex with 11nt-ssDNA, taken from the cryo-EM structure of the human core TFIIH complex with the DNA (PBD ID 6RO4 [17]). XPD protein includes four domains: Arch, FeS, ATPase lobe 1, and ATPase lobe 2, color coded and labelled according to constituent subunits. XPD has a central tunnel, accommodating ssDNA with its 3'- and 5'-ends toward the DNA entry and exit pore, respectively. Only 11nt-ssDNA (purple-blue strand) from Chain B with residues 32−42 are shown. The iron-sulfur cluster (FeS cluster) adjacent to the DNA entry pore is indicated as orange and yellow spheres, respectively. A view into the DNA entry pore, indicated with a red rectangular box, is formed by the FeS, Arch, and ATPase lobe 1 domains.

Zoom-in view into the DNA entry pore. Top panel, the surface rendered XPD protein has a DNA entry pore where the 3'-end ssDNA passes through. Nucleotides outside the pore are not visible in this cryo-EM structure. In proximity to the entry pore, there is a deep pocket comprised of residues Y192 and R196 together with aromatic residues Y158, F161, and F193, as depicted in (B). Bottom panel, the Arch domain residue R380 is in close contact with the ATPase lobe 1 helix containing residues 215−221, forming a closed state of the pore to encircle the 3'-end nucleotide dT (3'dT) (details see **Supplementary Figure S2A**).

(B) In cryo-EM structure with PBD ID 6RO4, zoom-in view into the DNA entry pore depicts the interactions between XPD-ssDNA near the entry pore. Only the last two 3'-end nucleotides, dA0 and dT1, accommodated within the entry pore, are displayed for clarity. Note that the DNA abbreviations, “dA0” and “dT1”, indicate the nucleotide “dA” at position 0 and “dT” at position 1, respectively; these correspond to Chain B residues 41 and 42 in PBD ID 6RO4 [17]. The XPD residues surrounding these two nucleotides are shown with sticks and color coded according to their constituent domains. The phosphate and sugar moieties of d(AT) are bound to the FeS residues Y192, R196, and R112 as well as the ATPase lobe 1 residue Y211. R112 and C134 bridge the DNA to the FeS cluster. **Inset box**, schematic representation of interactions of XPD with the last two 3'-end nucleotides, dA0 and dT1.

(C) Initial models of XPD in complex with lesion-containing and lesion-free ssDNA.
In **Model 1**, the last 3'-end nucleotide dT (3'dT, purple-blue) of the 11nt-ssDNA bound to XPD in PDB ID 6RO4 was removed and d(<u>TT</u>TG) (underscore denotes the 6−4PP lesion, colored in red) were modeled in at positions (1, 2, 3, 4). Bottom panel, surface rendered XPD protein shows that the bases of the 6−4PP lesion are positioned right *outside* the edge of the entry pore, named **XPD-lesionOUT**.

In **Model 2**, the last two 3'-end nucleotides (dT1 and dA0, purple-blue) bound to XPD in PDB ID 6RO4 were removed and d(<u>TT</u>TG) were modeled in at positions (0, 1, 2, 3). The 5T and 3T of the 6−4PP lesion were placed at the positions where the dA0 and dT1 are in PDB ID 6RO4, respectively. Bottom panel, surface rendered XPD protein shows that both bases of the 6−4PP lesion are positioned *within* the entry pore, named **XPD-lesionIN**.

In **Model 3**, the 3'-end nucleotide dT (3'dT, purple-blue) in PDB ID 6RO4 was removed and d(TTTG) in B-form was modeled into the positions (1, 2, 3, 4), named **XPD-unmod.** Bottom panel, when compared to 3'dT in PDB ID 6RO4, the modeled dT at position 1 (dT1) is reoriented toward the ATPase lobe 1 domain so that the extended B-form d(TTTG) can extend near the entry pore without causing steric clashes with the XPD residues. Furthermore, dT1 is shifted outside the pore, creating an unoccupied binding site within the pore (for details see **Supplementary Figure S2B**).

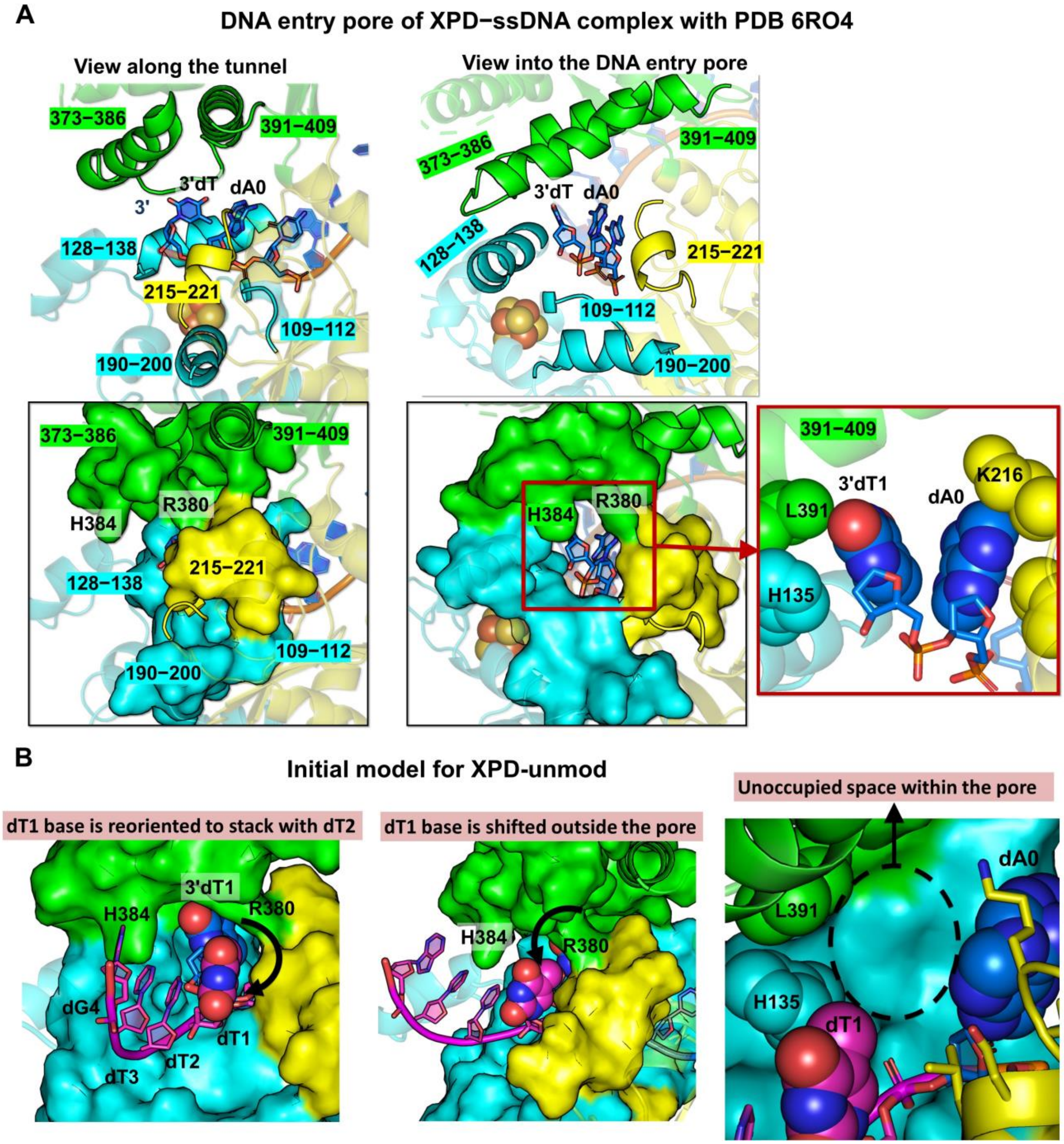

Supplementary Figure S2. Overview of the DNA entry pore in the XPD−11nt-ssDNA complex in PBD ID 6RO4 and the initial model with the DNA extension used for the simulation of XPD-unmod.

(A) Cryo-EM structure of XPD−ssDNA complex with PBD ID 6RO4 [17] does not include the extended DNA protruding outside the entry pore and the DNA binding sites within the entry pore are fully occupied. Two views near the entry pore show that the binding sites occupied by the last two nucleotides (3'dT1 and dA0) at the 3'-end are encircled by the XPD helices, including the FeS residues 109−112, 128−138, 190−200 (cyan), the Arch residues 373−409 (green), and the ATPase lobe 1 residues 215−221 (yellow). The side-chains of the Arch

residues R380 and H384 are hanging right outside the entry pore. A zoom-in view shows that the two bases of 3'dT1 and dA0 are stacked and housed between H135 (FeS), L391(Arch) and the ATPase lobe 1 residues K216 and I217, leaving no unoccupied space within the entry pore.

(B) Initial model of XPD-unmod includes an incoming ssDNA extended outside the entry and contains an unoccupied space within the entry pore. To construct an initial model for XPD-unmod that contains an unoccupied space within the entry pore and an incoming ssDNA extended outside the entry without causing any steric clash with the side-chains of R380 and H384, we utilized a B-DNA with sequence dTTTG (numbering 1, 2, 3, 4, respectively) outside the entry pore and replaced 3'dT1 in PDB 6RO4 with dT1 of the extended DNA (left and middle panels). **Left panel,** we retained the phosphate moiety of the 3'dT1 and then manually oriented the base and sugar of dT1 toward the ATPase lobe 1 helix so that its T base can stack with its 3'-nucleotide dT2 in B-form without causing steric clash with the residues R380 and H384. **Middle panel**, the dT1 base of the extended DNA is shifted outside the pore compared to 3'dT1in cryo-EM structure. **Right panel**, a zoom-in view shows an unoccupied space between H135, L391, and the base of dA0 within the entry pore in the initial model of XPD-unmod. Overall, this initial model of XPD-unmod contains an unoccupied space within the entry pore and an incoming extended DNA protruding outside the entry; thus, it is a translocation-capable model.

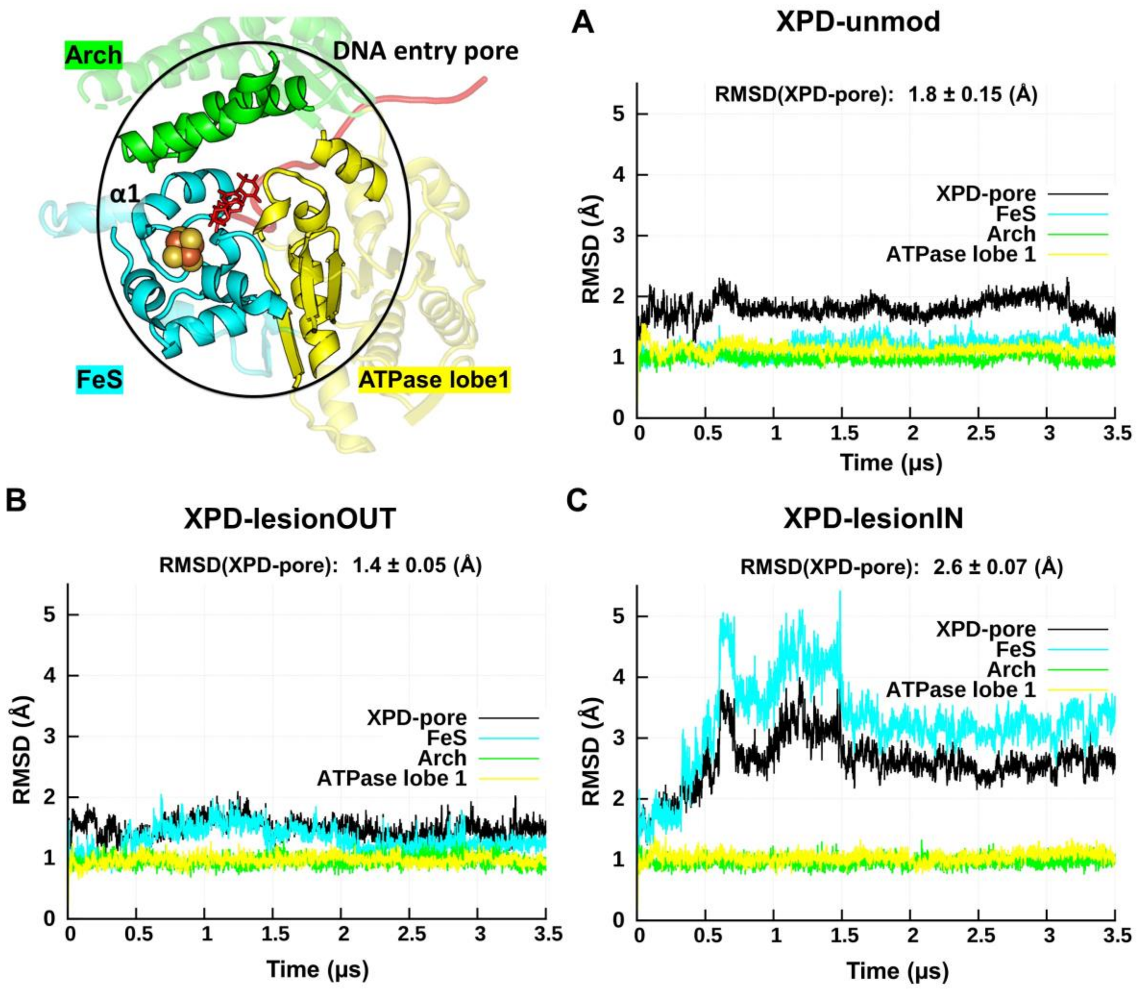

Supplementary Figure S3. XPD retains a correctly folded pore when binding to ssDNA regardless of the presence or absence of the lesion.

We computed the time-dependent backbone Cα RMSDs of the XPD pore as indicated in black circle and the Cα RMSDs of the individual domains (Arch, FeS, and ATPase lobe 1) constituting this entry pore. Overall, after 1.5 μs in all XPD cases run to 3.5 μs, the XPD pore reaches a stable conformation and retains a correctly folded structure with RMSDs of ~ 1.4 − 2.6 Å from its initial fold. Mean values and standard deviations of the RMSDs of the XPD pore from the equilibrium ensemble (2 − 3.5 μs) are given.

(**A**) In **XPD-unmod**, the RMSD of the XPD pore reaches a stable value after ~ 0.8 μs.

(**B**) In **XPD-lesionOUT**, the XPD pore reaches a stable conformation after ~ 1.5 μs.

(**C**) In **XPD-lesionIN**, the RMSDs of the XPD pore are more deviated from the initial fold compared to **XPD-unmod** and **XPD-lesionOUT**. The mobility of the XPD fold is mainly dominated by the FeS domain near the pore, as reflected in the correlation in their RMSDs (for details see **Supplementary Figure S4)**. After reaching the stable state at ~1.5 μs, the XPD pore still retains a correctly folded structure with RMSD of 2.6 ± 0.07 Å.

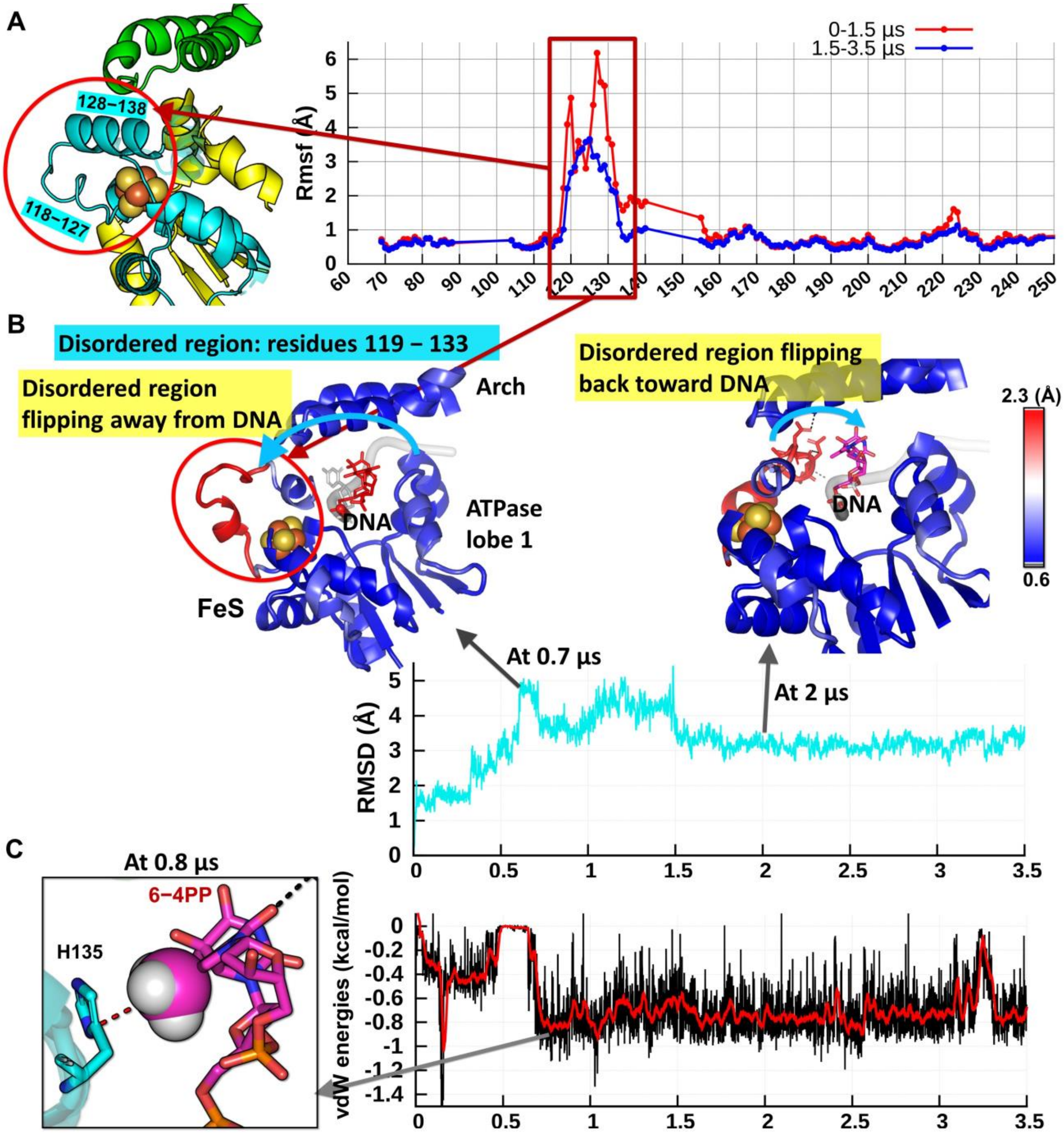

Supplementary Figure S4. FeS residues 119−133 are responsible for the dynamics of the XPD pore prior to equilibration in XPD-lesionIN.

In **XPD-lesionIN**, the RMSDs show that the XPD pore exhibits the greatest deviation from its initial fold (with ensemble average RMSDs of ~ 2.6 Å) and is the most dynamic, particularly during the 0.3 to 1.5 µs interval (**Supplementary Figure S3C**). This mobility of the XPD fold is mainly dominated by the FeS residues 119–133, which are located within a loop (residues 118−127) and a helix (residues 128−138) near the FeS cluster. During the 0.3 ~ 1.5 µs interval, the FeS helix is partially flipped away (residues 128−133) from the DNA and disordered. At ~ 1.5 µs, the side chains of residues K128, D129, and D131 are flipped back toward the DNA,

which reduces the dynamics thereafter. We noted that despite the existence of this partially disordered helix, beginning at ~ 0.8 μs, the aromatic residue H135 forms stable close contacts with the methyl group of the lesion 3T base via methyl-π interactions, narrowing the pore width as reflected in the shortened Cα(H135)−Cα(L220) distance (XPD-lesionIN in **Supplementary Figure 7B**); this facilitates the immobilization of the lesion within the pore. The movie of **XPD-lesionIN** (**Supplementary MovieS4**) reveals that the modified 3T base of the lesion is tightly-squeezed by the Arch, the FeS, and the ATPase lobe 1 domains; this immobilization of the lesion is not affected by the presence of the partially disordered region (residues 128−133) in the FeS helix.

(**A**) Analysis of the root mean square fluctuations (RMSFs, indicative of individual residue flexibility) of the XPD pore revealed that the FeS residues 119–133, which are located within a loop (residues 118−127) and a helix (residues 128−138) near the FeS cluster, are entirely responsible for these highly fluctuating RMSDs during the 0.3−1.5 μs interval in **XPD-lesionIN** (**Supplementary Figure S3C**).

(**B**) The FeS helix is partially flipped away (residues 128−133) from the DNA and becomes disordered during the 0.3 ~ 1.5 μs interval (see structure at 0.7 μs). At ~ 1.5 μs, the side chains of residues K128, D129, and D131 are flipped back toward the DNA, reducing the dynamics thereafter (see structure at 2 μs).

(**C**) Beginning at ~ 0.8 μs, the aromatic residue H135 forms stable close contacts with the methyl group of the lesion 3T base via methyl-π interactions, narrowing the pore width as reflected in the shortened Cα(H135)−Cα(L220) distance (**Supplementary Figure S7B).**

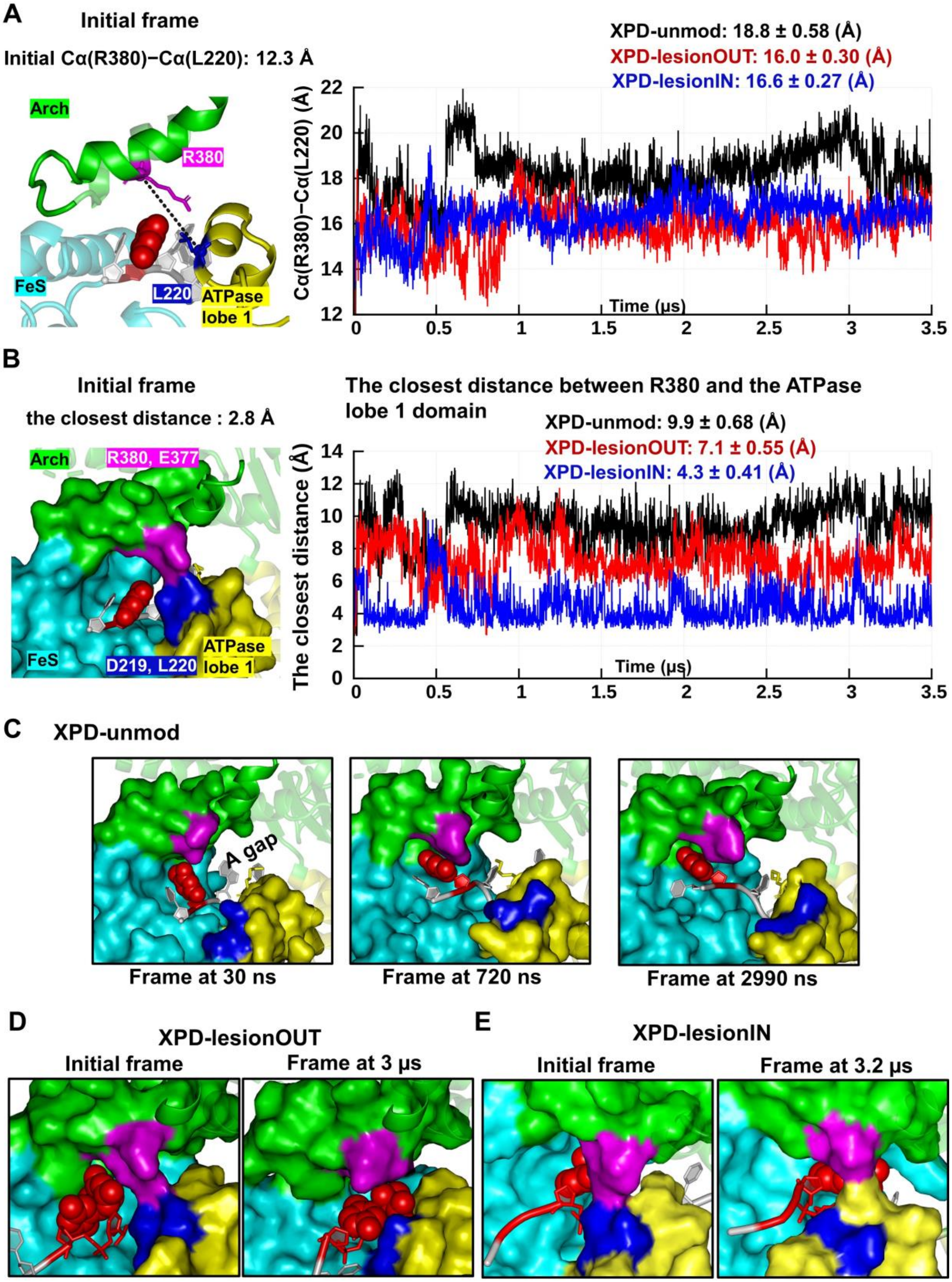

Supplementary Figure S5. Right at the edge of the DNA entry pore, there is a noticeable gap between the Arch and the ATPase lobe 1 domains for the undamaged DNA, while in the lesion-containing XPDs, this gap is either negligible or absent**.**

In the initial structure for all XPD complexes, right at the entry, the side chain of R380 points toward the ATPase lobe 1 helix containing residues 215 − 221, particularly D219 and L220, resulting in a closed state of the pore. Notably, we observed that a gap is generated in this region in the case of **XPD-unmod**. However, the gap is negligible in the lesion-containing XPDs and nearly absent in the case of **XPD-lesionIN**. The gap is attributed to the increased distance between the Arch and the ATPase lobe 1 domains, as well as the increased distance between the side chain of R380 and the ATPase lobe 1 helix residues. We measured the distance between Cα(R380) of the Arch and Cα(L220) of the ATPase lobe 1 domain, as an indicator of the relative displacement of the Arch domain from the ATPase lobe 1 domain. At the initial frame, the distance Cα(R380)−Cα(L220) is ~ 12.3 Å. We also monitored the closest distance of R380 heavy atoms to the ATPase lobe 1helix, as an indicator of the closed or opened state of the pore. At the initial frame, this distance is ~ 2.8 Å in the closed state of the pore. In all structures presented, right at the entry pore, the Arch residues E377 and R380 are highlighted as magenta and the ATPase lobe 1 residues D219 and L220 in blue to visualize the conformational change of the entry pore in the presence of undamaged DNA and lesion-containing DNA.

(**A**) Time dependence of the distance between Cα(R380) of the Arch residue and Cα(L220) of the ATPase lobe 1 residue, as an approximate indicator of the relative position of the Arch domain with respect to the ATPase lobe 1 domain. Their mean values and standard deviations from the equilibrium ensemble are given.

(**B**)Time dependence of the shortest distance of the heavy atoms of R380 to the lobe 1 helix (residues 215−221), as an indicator of the closed or open state of the pore. Their mean values and standard deviations from the equilibrium ensemble are given.

(**C**) In **XPD-unmod**, early in the simulation (Frame at 30 ns), the Arch domain is shifted away from the ATPase lobe 1 domain and the side chain of R380 points away from the ATPase lobe 1, as reflected in the increased Cα(R380) − Cα(L220) distance, by ~7 Å compared to the initial frame. These rearrangements cause a notable gap at the entry pore, with significantly increased distance between R380 and the ATPase lobe 1 domain, by up to ~ 9 Å compared to the initial frame, producing an open state of the pore. This gap persists throughout the simulation and is dynamic. Note that the ATPase lobe 1 also releases its hold on the base of the undamaged dT1, which is thus oriented toward the interface between the Arch and the FeS domains.

(**D**) In **XPD-lesionOUT**, at the initial frame the 6−4PP lesion (red sphere) is positioned right outside the entry pore, which is in the closed state. During the equilibrated state (frame at 3 μs), there is a small gap generated between the Arch and the ATPase lobe 1 domains at the entry pore. However, this gap is only just large enough to accommodate the 5T base of the lesion so that it is clamped tightly between the Arch and the ATPase lobe 1 domains at the pore.

(E) In **XPD-lesionIN**, the 6−4PP lesion is initially positioned at the entry pore, which is in the closed state. During the equilibrated state, the lesion remains encircled by a closed, narrow pore with its 3T base tightly bound to the XPD domains.

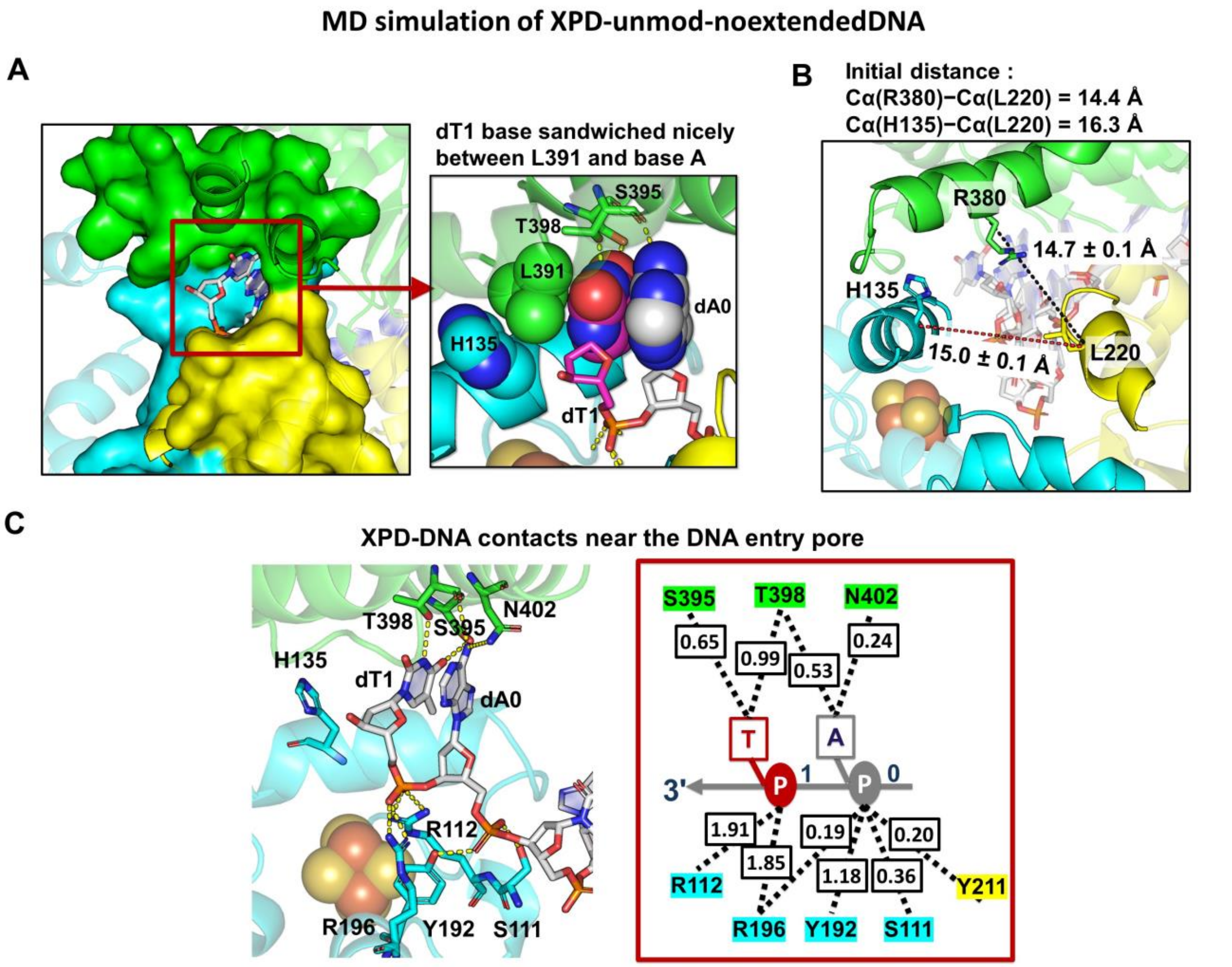

Supplementary Figure S6. MD simulation of XPD-unmod-noExtendedDNA.

The most representative structure from the equilibrate ensemble (during 1.5 – 2 μs) is displayed.

(A) A view along the entry pore (left and middle panels) shows that the last two 3'-end nucleotides dT1 and dA0 are positioned well within the pore. **Right panel**: a zoom-in view shows that the base dT1 is sandwiched between L391 and its 5'−A base, and its T base forms hydrogen bonds with T398.

(**B**) A view into the entry pore showing that near the entry, the Arch domain retains its relative position with the ATPase lobe 1 domain, reflected in ensemble average Cα(R380)−Cα(L220) distance of ~ 14.7 Å, similar to its initial value.

(C) Left panel, hydrogen bonds (yellow dashed lines) between the nucleotides dT1 and dA0 and their surrounding XPD residues. The ATPase lobe 1 domain is not displayed for clarity. **Right panel** shows schematic representation of these hydrogen bonds.

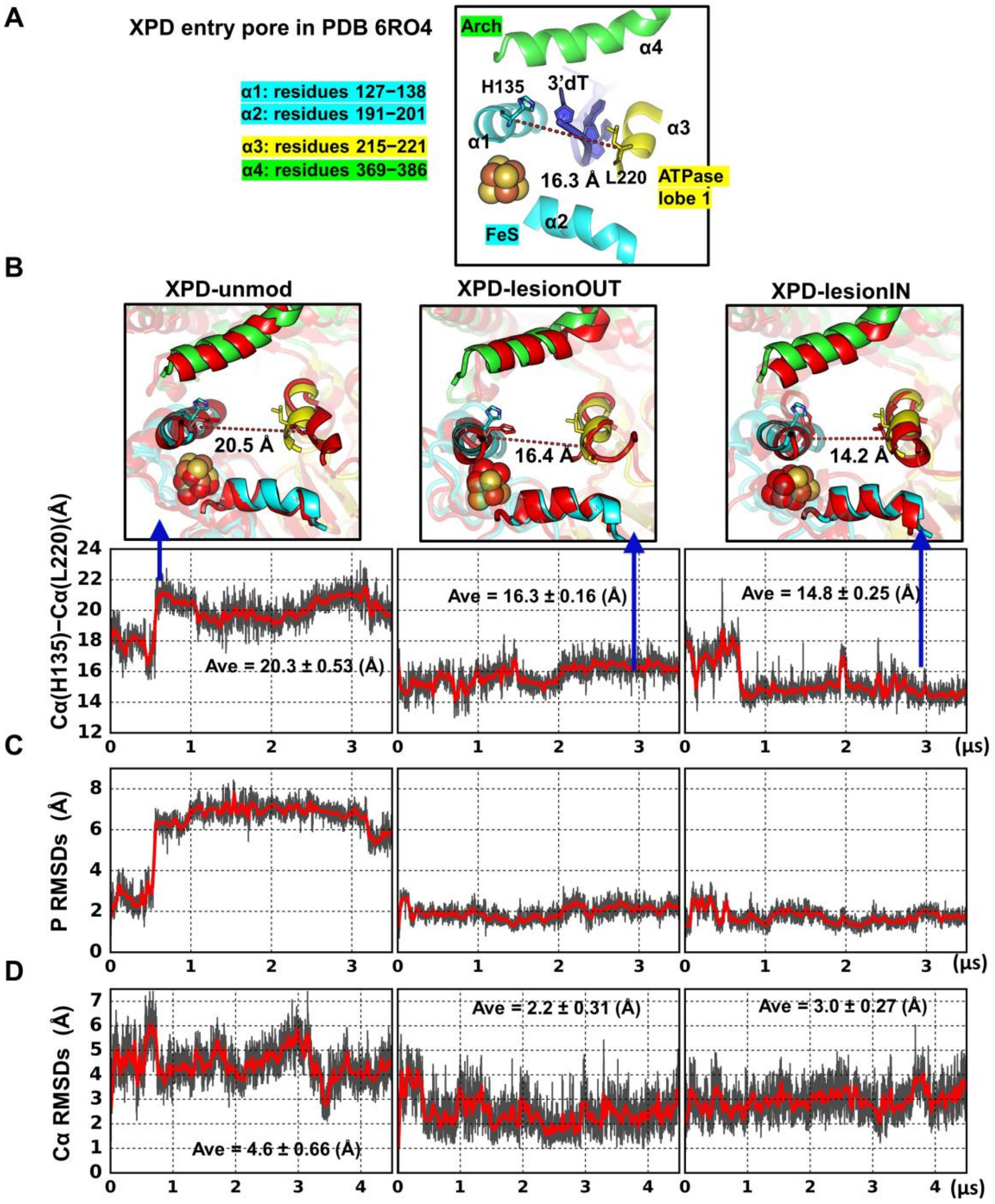

Supplementary Figure S7. In lesion-free XPD, the unmodified DNA undergoes a backbone translocation through the XPD pore; the pore is greatly enlarged and is more dynamic than in the lesion-containing XPDs.

(**A**) In the cryo-EM structure with PBD ID 6RO4, there are four helices (α1−α4) from each domain surrounding the entering 3'-end ssDNA. We measured the distance between Cα(H135) of the FeS and Cα(L220) of the ATPase lobe 1 domain with a value of 16.3Å, as an indicator of the pore width. The Arch, the FeS, and the ATPase lobe 1 domains are color coded and labelled according to constituent subunits. The DNA is colored as purple-blue.

(**B**) Time dependence of the Cα(H135)−Cα(L220) distance for each XPD complex. Mean values and standard deviations from the equilibrium ensemble (between 2.0 and 3.5 μs) for all the XPDs are also given. Higher values in standard deviations indicate a more flexible pore. Structures shown are a comparison of simulated XPD pore (colored as red) with the one in PDB ID 6RO4 (color coded as displayed in A). In **XPD-unmod**, the ensemble average value of Cα(H135)−Cα(L220) of ~ 20.3 ± 0.53 Å is the greatest; this large value is mainly due to the enlarged pore width that results from the ATPase lobe 1 helix with residues 215 − 221 tilting away from the center of the XPD pore. In **XPD-lesionOUT**, the pore width is close to that in PDB ID 6RO4. In **XPD-lesionIN**, the pore width is the narrowest; this is mainly because the FeS helix containing residues 128−138 has tilted closer to the center of the pore (see **Supplementary Figure S4C**). The structures displayed are the most representative ones from the equilibrated ensemble in each XPD complex.

(**C**) Time-dependence of the P atom RMSDs for the 6−4PP lesion in the lesion-containing XPDs and for the dT1 in the lesion-free XPD was monitored. In **XPD-unmod**, time-dependent P atom RMSDs of the unmodified dT1 reveal a rapid jump from ~ 3Å to ~ 6.2 Å at 0.5 μs, reflecting the backbone translocation of the unmodified dT1 passing through the pore. This is well-correlated with the greatly enlarged distance of Cα(H135)−Cα(L220) at this time; this correlation shows that the Cα(H135)−Cα(L220) distance is a good indicator of the pore size, which is large enough to permit a nucleotide to pass through. On the other hand, for the lesion-containing XPDs, the backbone of the bulky lesion is nearly immobilized and the XPD pore is much smaller and less dynamic than in the lesion-free XPD.

(**D**) Relative motion of the Arch and the FeS domains near the entry pore, which are obtained by monitoring the Cα RMSD of the Arch domain after fitting the stable region of the FeS Cα atoms to the initial structure for each XPD-ssDNA case. In **XPD-unmod**, the Arch domain deviates from its initial structure by more than ~ 8 Å during the backbone translocation of dT1 shown in (C) and also during a rapid increase in the Cα(H135)−Cα(L220) distance shown in (B); thus, these suggest that the Arch shifts away from the ATPase lobe 1 domain (**Supplementary Figure S5A)**. The Arch domain also exhibits much greater fluctuations in the RMSD here than in the lesion-containing cases. These results indicate that in the presence of the undamaged DNA, the Arch domain is more mobile with respect to the FeS domain than in the presence of the lesion.

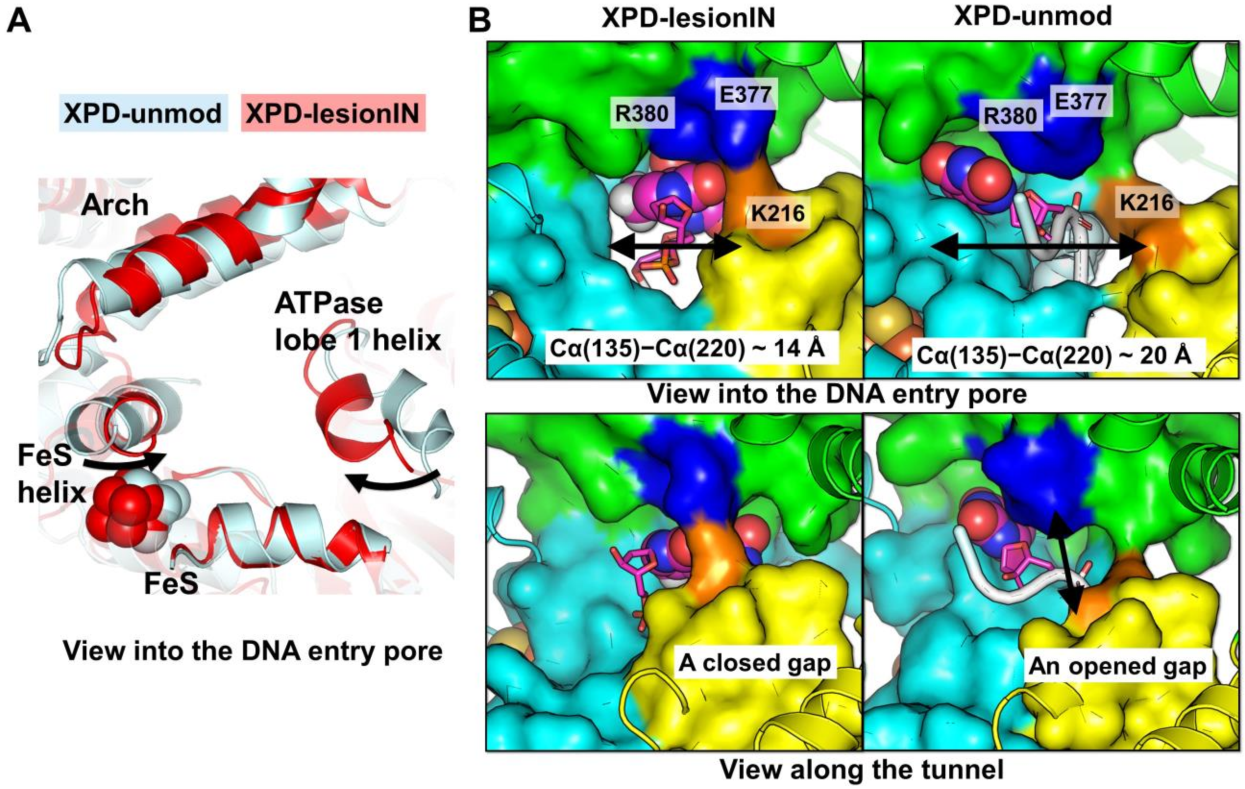

Supplementary Figure S8. XPD pore is opened for the unmodified DNA while closed for the damaged DNA, mainly because the ATPase lobe 1 helix releases its grip on the undamaged DNA and points away from it.

(**A**) Two aligned structures, one with the lesion within the pore (**XPD-lesionIN**, red) and another with the unmodified DNA (**XPD-unmod**, light-cyan); alignment is based on the FeS domain. A view into the entry pore illustrates that the ATPase lobe 1 helix releases its grip on the undamaged DNA and points away from it, enlarging the XPD pore width. In contrast to the undamaged DNA, when encountering a lesion within the pore, both ATPase lobe 1 and FeS helices are closer to the damaged DNA, narrowing the pore width.

(**B**) Two views illustrating that the modified bases are held tightly within a narrow pore with a closed gap between the Arch (E377and R380) and the ATPase lobe 1 (K216) helices, while the undamaged dT1 base is in an enlarged pore with a noticeable gap between the Arch and ATPase lobe 1 helices. In **XPD-lesionIN**, the modified bases are held stably by all three domains, indicating that the Arch domain is locked down on the ATPase lobe 1 or the FeS domains. However, in XPD-unmod, the undamaged dT1 base is positioned at the interface between the FeS and the Arch domain with E377 and R380 pointed toward the DNA; furthermore, the ATPase lobe 1 helix is tilted away from the DNA; as a result, there is a noticeable gap near the entry pore.

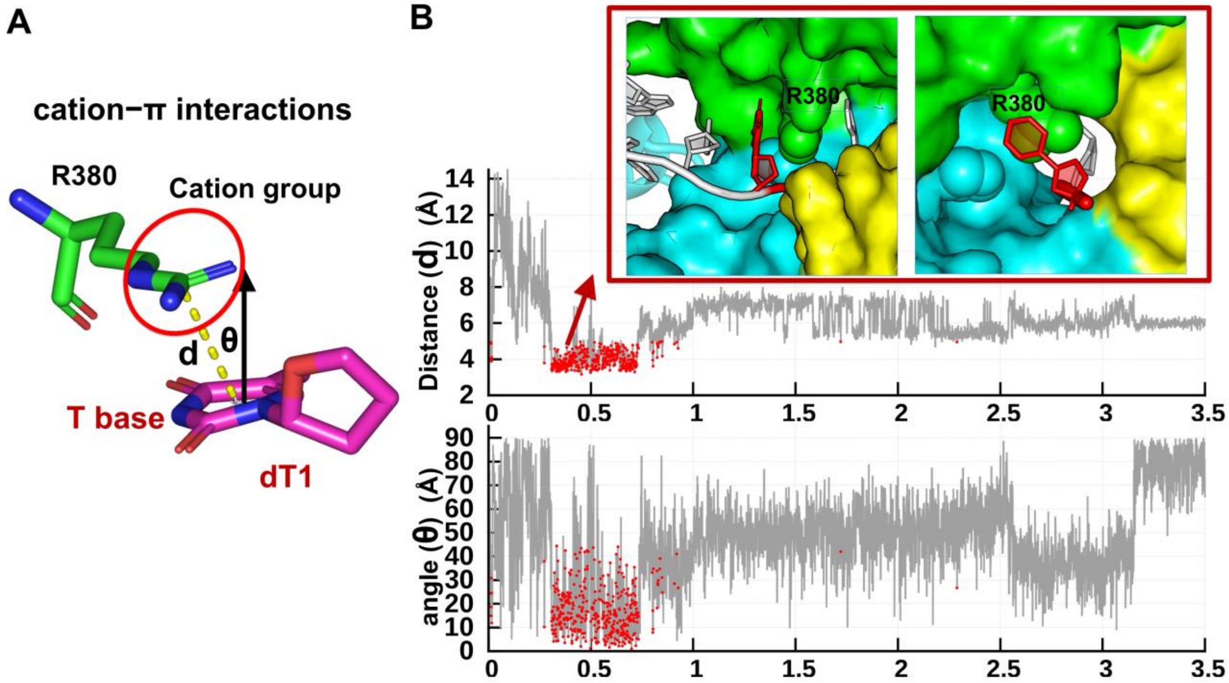

Supplementary Figure S9. A stacked cation-π interaction between a native T base and a guanidium side chain of R380 in XPD-unmod.

(A) Cation-π interactions are detected by two parameters [18]: distance (**d**) between the planes of the cationic group of R380 and the planar group of the T base (d < 5 Å), and angle (**θ**) between them (θ < 45°).

(B) Time-dependent distance (**d)** and angle (**θ)** show that the cation-π interaction between R380 and the native base of dT1occured mainly during 0.3 − 0.7 µs (prior to equilibration); these interactions pulled the dT1 base away from the ATPase lobe 1 and toward the interface between the Arch and FeS domains; hence the dT1 base can be flipped into the pore, as it is sandwiched between H135(FeS) and R380 (Arch), as shown in **Figure 3D**. Furthermore, there is no stable cation-π interaction found in the lesion-containing XPDs (data not shown). Thus, stable cation-π interactions between R380 and DNA are not observed during the equilibrated state for any of the XPD-ssDNA cases, but this cation-π interaction is a component of the process for translocating the dT1 into the pore prior to equilibration.

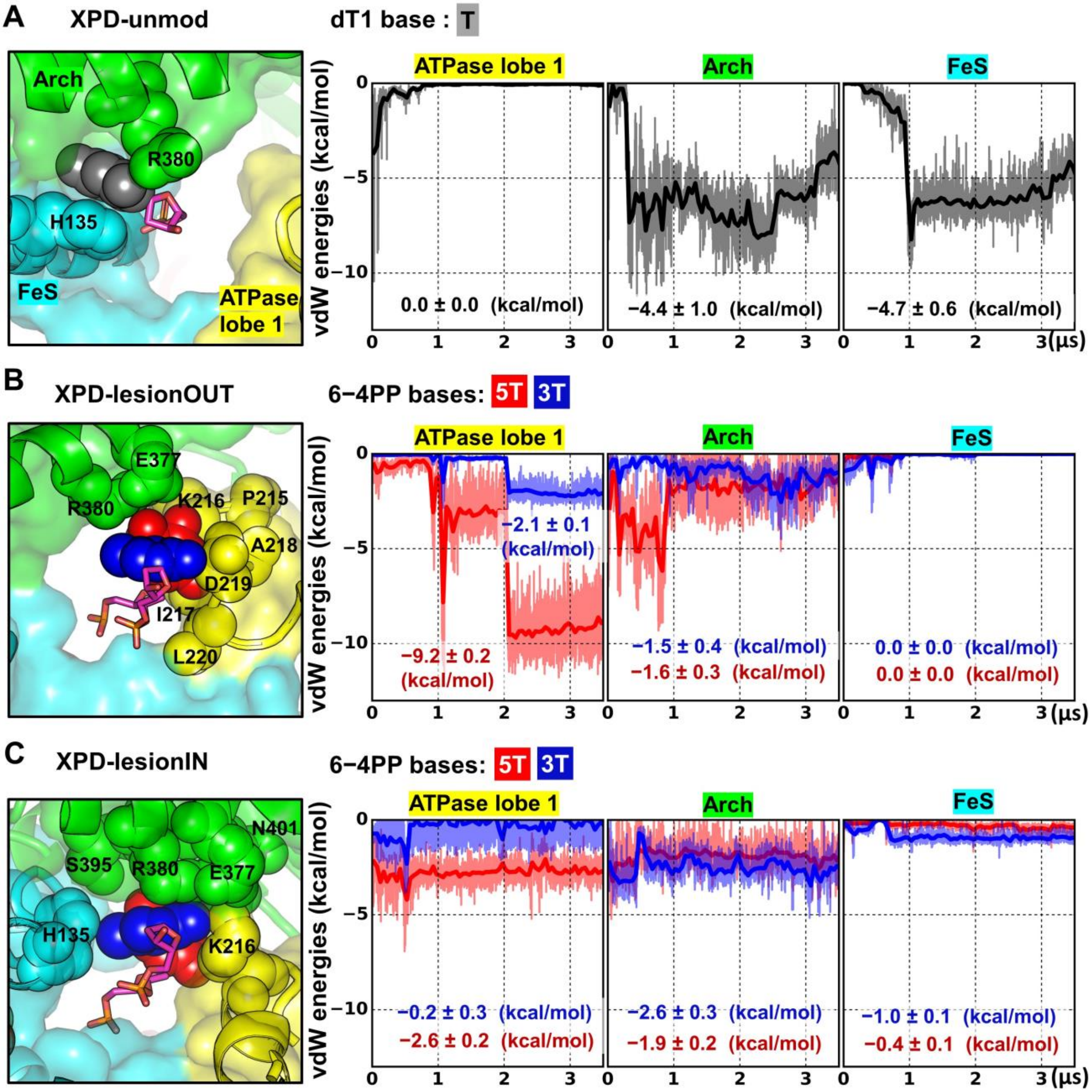

Supplementary Figure S10. The bases of the 6−4PP lesion form van der Waals interactions with the ATPase lobe 1 domain, while these interactions are completely absent in the case of lesion-free XPD, because the unmodified base T points away from the ATPase lobe 1 domain and points toward the interface between the interface between the Arch and the FeS domains.

We computed the time dependence of the van der Waals interaction energies of the XPD pore (ATP lobe 1 residues 215−221, the Arch residues 369−409, and the FeS residues 128−138) with the unmodified base T (gray lines) in the lesion-free case and with the individual bases 5T (red lines) and 3T (blue lines) of the 6−4PP in the lesion-containing cases. These van der Waals interactions are the manifestation of the orientation of the unmodified base within the pore in the lesion-free case or the modified bases in the lesion-containing cases. Mean values and standard deviations are computed based on the equilibrium ensemble for all the XPDs. The most

representative structures from the equilibrium ensemble are also displayed for each XPD, illustrating the orientation of the unmodified base T and modified bases (5T and 3T) within the entry pore. In all structures presented, each domain of the XPD pore is color coded and labelled according to constituent subunits. The key residues that contribute to the van der Waals interactions with the T base in the lesion-free case or the modified bases 5T and 3T in the lesion-containing cases are labeled and rendered as spheres. In **XPD-unmod**, the T base is shown as gray sphere, and in the lesion-containing XPDs, the bases 5T and 3T of the 6−4PP lesion are shown as red and blue spheres, respectively.

(**A**) In **XPD-unmod**, the T base is initially oriented toward the ATPase lobe 1 domain. After 1 µs, the T base is re-oriented toward the interface between the Arch and the FeS domains where it is sandwiched stably between H135(FeS) and R380(Arch) (also see **Figure 3D**) during 1 − 3 µs and its van der Waals interactions with H135(FeS) persist throughout the simulation (also see **Figure 3D-E**). This completely abolishes the base's van der Waals interactions with the ATPase lobe 1 domain. In the equilibrium ensemble (between 2 − 3.5 µs), the van der Waals interactions of the base T with the Arch domain are dynamic, as reflected in the greater standard deviations.

(**B**) In **XPD-lesionOUT**, during the equilibrated state (after 2 µs), the bases 5T and 3T of the 6−4PP lesion are clamped tightly between the Arch and the ATPase lobe 1 domains. Notably, the modified 5T base forms stable van der Waals interactions with the lobe 1 residues 215 − 221 and the Arch residues E377 and R380 (**Figure 4B-C**).

(**C**) In **XPD-lesionIN**, the lesion is initially positioned within a closed pore and its bases are confined by all three XPD domains, as manifested by van der Waals interactions. These interactions persist throughout the simulation and are stable after ~ 0.8 µs. The bases of the lesion form van der Waals interactions mainly with K216 (ATPase lobe 1), E377, R380, S395, T398, and N351 (Arch), and H135 (FeS) (also see **Figure 5B**). Furthermore, the interactions between H135 (FeS) and the 3T base of the lesion pull the FeS helix that contains H135 closer toward the ATPase lobe 1 domain (**Supplementary Figure S4C**), explaining the narrowed pore width observed in **Supplementary Figure S7B**.

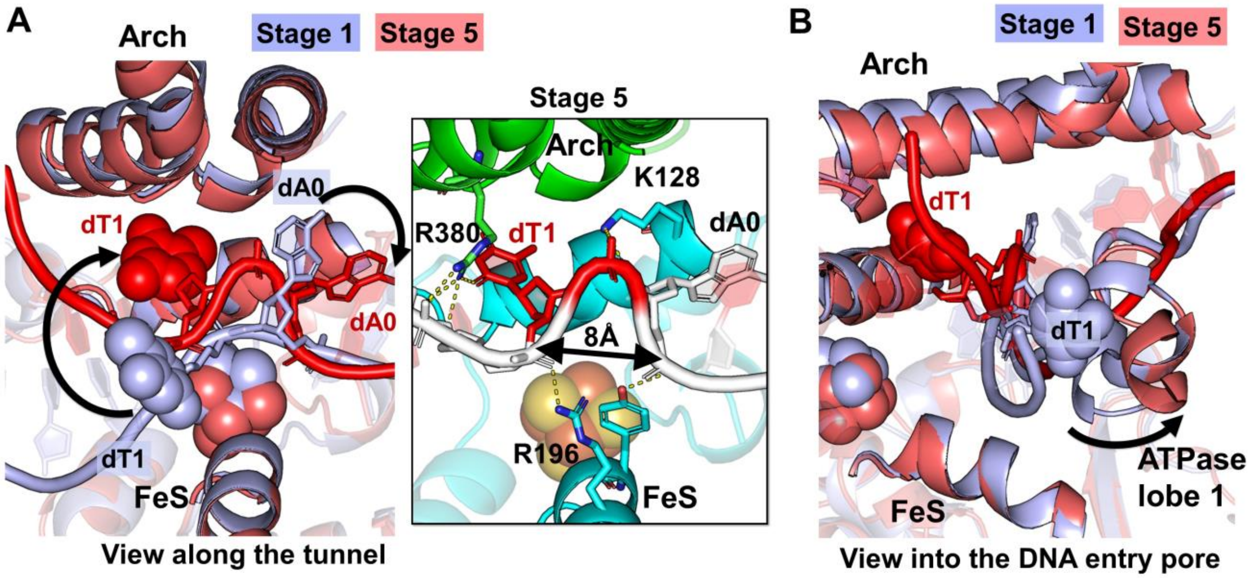

Supplementary Figure S11. Superimposed structures with dT1 prior to translocation (light-blue) and with dT1 after translocation (red).

We have superimposed the structures from **stage 5** where there is a DNA bend between dT1 and dA0 (**Figure 3E**) within the pore and from **stage 1**during 0 − 0.3 µs (prior to the process of dT1 translocation in **Figure 3A**). We did not find that noticeable displacement of the Arch domain, which is lifted away from the DNA, contributes to the extra space for the DNA bend between the dT1 and dA0. However, we did observe the base of dA0 pointing away from the position between residues 216 – 217 of the ATPase lobe 1 helix and the FeS helix (128 – 138) within the pore; this creates the space for the DNA bend between dT1 and dA0 as well as for the side chain of K128 that are pointed to the backbone of dT1. Furthermore, we also observed displacement of the ATPase lobe 1 helix (residues 215 − 221) away from the DNA, enlarging the pore width and aiding in the DNA to enter the pore.

(**A**) A view along the entry pore showing that during the dT1 transition, dA0 is pointed away from the position between the ATPase lobe 1 helix and the FeS helix within the pore; this creates the space for the DNA bend/loop between dT1 and dA0 as well as for the side chain of K128 pointed to the backbone of dT1 (zoom-in view). The ATPase lobe 1 domain is not shown for clarity. Zoom-in view shows that the basic residues K128, R196, and R380 bind to the phosphate backbone of this DNA bend to balance out the negative repulsive energy induced by the shortened phosphodiester distance of ~ 8Å across the loop.

(**B**) A view into the entry pore shows that during the dT1 transition, we did not find any displacements of the Arch or the FeS domains that moved them away from the DNA to create the extra space for the DNA bend; however, we observed a noticeable displacement of the ATPase lobe 1 helix (residues 215 − 221), which is tilted away from the DNA, and enlarges the pore width to allow the DNA to enter the pore.

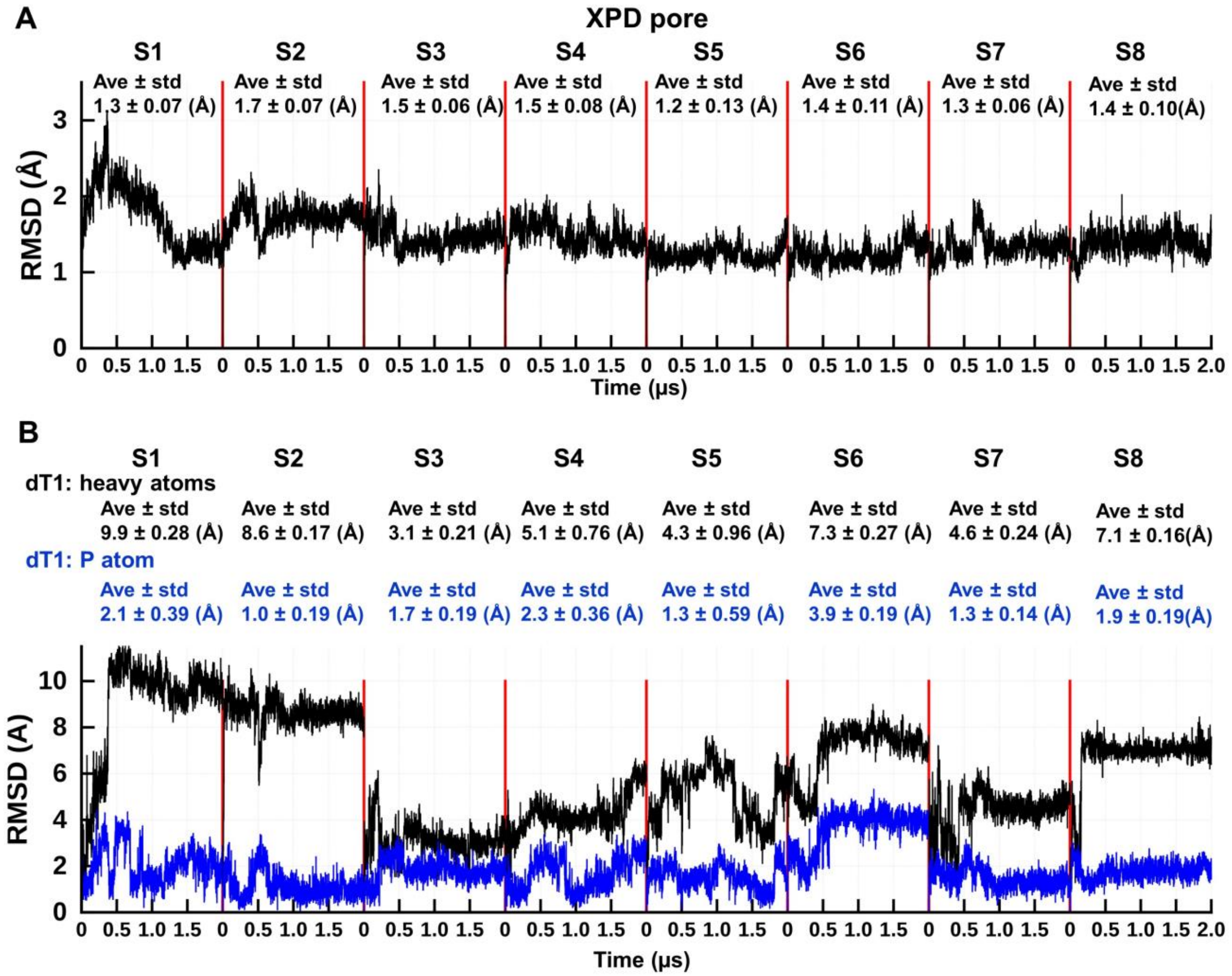

Supplementary Figure S12. XPD retains a correctly folded pore when binding to ssDNA in 8 additional independent MD simulations (S1 – S8) of ~ 2 μs each for lesion-free XPD.

(**A**) In these simulations, the time-dependent Cα RMSDs of the XPD pore reveal that after 1.5 μs, the XPD pore reaches a stable conformation and retains a correctly folded structure with RMSDs of ~ 1.4 − 1.7 Å from its initial fold. Mean values and standard deviations of the RMSDs of the XPD pore from the equilibrium ensemble (1.5 − 2.0 μs) are given.

(**B**) We computed the RMSDs of heavy-atoms and the backbone P atom of the undamaged dT1 in the lesion-free XPD while superimposing the XPD pore. The DNA reaches an equilibrated state after ~ 1.5 μs. These simulations show that during the equilibrated state, dT1 reveals a varying deviation from its initial conformation, with heavy atom RMSD value ranging from 3 to 10 Å. The deviation of dT1 is mostly due to its base reorientation as its phosphate backbone retains its position within the XPD pore, which is reflected in the P atom RMSD of 1.0 ~ 4 Å. Note that in all simulations, the phosphate backbone of dT1 is still anchored stably to R112 and R196. Mean values and standard deviations of the RMSDs of heavy-atoms and the backbone P atom of the undamaged dT1 during the equilibrium ensemble (1.5 − 2.0 μs) are given.

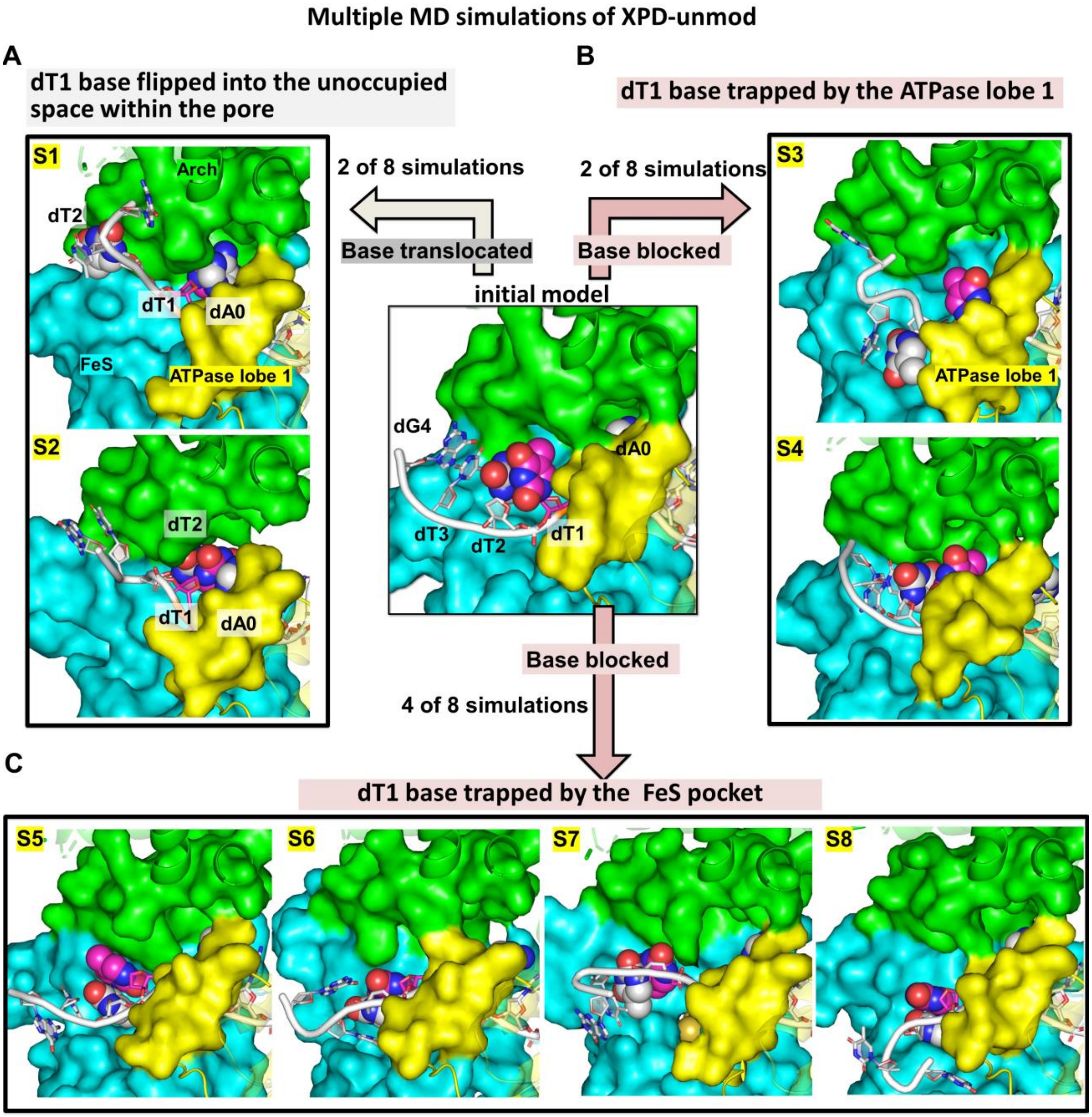

Supplementary Figure S13. Multiple MD simulations (8 x 2 µs) reveal that extended DNA is conformationally flexible.

The initial model of XPD-unmod contains an unoccupied space within the entry pore and an incoming extended DNA protruding outside the entry; thus, it is a translocation-capable model. These eight MD simulations revealed that dT1 displays varying behavior, with its base flipped into the unoccupied space within the pore (2 of these 8 simulations) and with its base blocked outside the pore (6 of these 8 simulations), indicating the highly flexible nature of the extended DNA and highlighting the base sensors near the entry (for details see **Supplementary Figure S14**). All structures displayed are the most representative structures based on the last 500 ns for each simulation. The bases of nucleotides dA0, dT1, and dT2 are shown as sphere. dT1 is

colored by atom with carbon atom in magenta and carbon atom in grey-white for other nucleotides.

(**A)** Two simulations revealed that either the one base of dT1 (in simulation S1) or the two bases of dT1 and dT2 (in S2) are flipped into unoccupied space and stacked with the base of dA0.

(B) Two simulations (S3 and S4) revealed that the dT1 base is captured by the ATPase lobe 1 helix (residues 215−22S1), inhibiting the base flipping into unoccupied space within the pore.

(C) Four simulations (S5−S8) revealed the dT1 base trapped by the hydrophobic pocket near the FeS cluster, blocking dT1 from entering the pore.

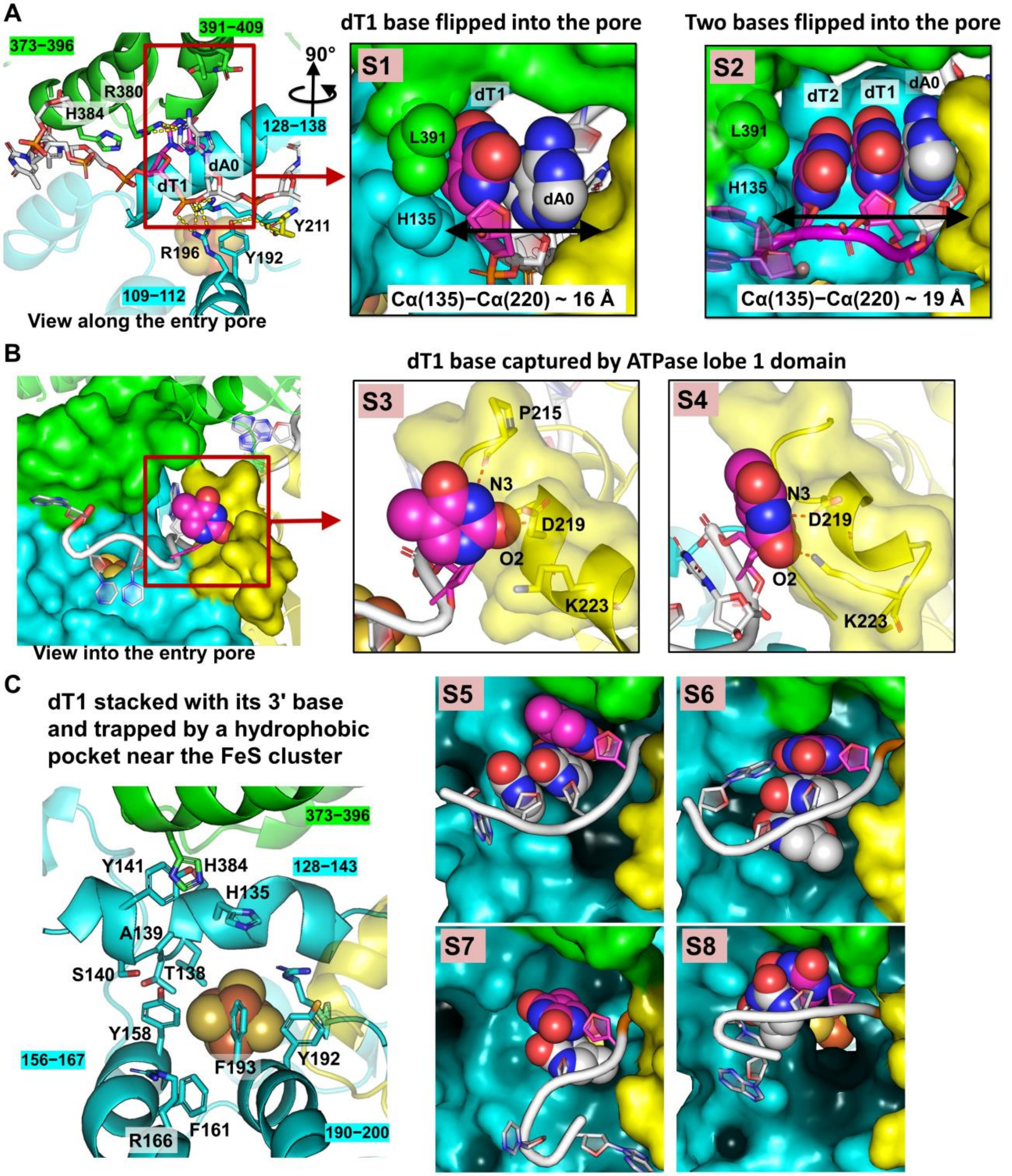

Supplementary Figure S14. Details of the varying behaviors of dT1 in the DNA extension from 8 independent MD simulations of XPD-unmod.

(**A**) Simulation **S1** revealed that the dT1 base is flipped into unoccupied space within the pore with its base stacked with L391, H135 and the base of dA0 as well as with R380 and H384 on its 3'-side and the rest of the extended DNA on the other side of R380 and H384, very similar to the

cryo-EM structure [17]. Furthermore, the pore width, Cα(H135)−Cα(L220) ~ 16 Å, is close to the cryo-EM structure with a value of 16.3Å. The phosphate moieties of dA0 and dT1 retain the interactions with R112, Y192, R196, and Y211. Thus, these similarities suggest that our MD simulations starting from our initial model may still reflect, while not in the major population, the conformation taken by the 3'-tail in the experiment.

Simulation **S2** revealed that the bases of dT1 and dT2 are flipped into the pore. The XPD pore width is opened with a value of 19Å, allowing two stacked bases of dT1 and dT2 to enter.

(**B**) Two simulations (**S3** and **S4**) revealed that the dT1 base is captured by the ATPase lobe 1 helix with residues 215−221 via two different orientations.

(**C**) Four simulations (**S5−S8**) revealed that the dT1 base is trapped into the pocket near the FeS cluster. This pocket, mainly composed of several hydrophobic residues (H135, A139, S140, Y141, Y158, F161, Y192, F193), can trap multiple bases (shown as spheres).

Supplementary Movies

Supplementary MovieS1.mp4: Simulation (0 − 1.2 µs) of **XPD-unmod** reveals that undamaged dT1 initially positioned outside the DNA entry pore undergoes a one-nucleotide 3'→5' translocation as it enters the pore.

Supplementary MovieS2.mp4: Best representative structure of **XPD-lesionOUT** during the equilibrated state (during 2 − 3.5 µs) reveals that the 6−4PP lesion is immobilized by the XPD; the modified 5T base is clamped between the Arch and the ATPase lobe 1 domains and its N3 and O2 atoms are captured by a small pocket formed by the ATPase lobe 1 helix, blocking its translocation into the pore.

Supplementary MovieS3.mp4: Best representative structure of **XPD-lesionIN** during the equilibrated state (during 2 − 3.5 µs) reveals that the 6−4PP lesion initially positioned at the DNA entry pore is immediately immobilized by the XPD; the modified 3T base is confined by all three XPD domains: FeS, Arch and ATPase lobe 1 during the equilibrated state, preventing it from translocating further into the pore.

Supplementary MovieS4.mp4: Simulation (0 − 2.0 µs) of **XPD-lesionIN** reveals that the modified 3T base of the 6−4PP lesion is tightly-squeezed by the Arch, the FeS, and the ATPase lobe 1 domains beginning at ~ 0.8 µs; this immobilization of the lesion is not affected by the presence of the partially disordered region (128 − 133) in the FeS helix (128 − 138) that is particularly mobile during 0.3 − 1.5 µs interval.

Supplementary MovieS5.mp4: Simulation (0 − 2.5 µs) of **XPD-lesionOUT** reveals how the modified bases of the lesion transit from state 1 (0 − 1 µs) to state 2 (1 − 2 µs), and to the equilibrated state (2 − 2.5 µs); in the latter, the modified 5T of the 6−4PP lesion is clamped between the Arch (E377 and R380) and the ATPase lobe 1 (residues 215 − 221) with its N3 and O2 atoms captured by a small pocket formed by the ATPase lobe 1 helix.

Supplementary References

[1] J.A. Maier, C. Martinez, K. Kasavajhala, L. Wickstrom, K.E. Hauser, C. Simmerling, ff14SB: Improving the cccuracy of protein side chain and backbone parameters from ff99SB, J. Chem.Theory Comput. 11 (2015) 3696-3713.

[2] D. Paul, H. Mu, H. Zhao, O. Ouerfelli, P.D. Jeffrey, S. Broyde, J.H. Min, Structure and mechanism of pyrimidine-pyrimidone (6-4) photoproduct recognition by the Rad4/XPC nucleotide excision repair complex, Nucleic Acids Res 47 (2019) 6015-6028.

[3] A.T.P. Carvalho, M. Swart, Electronic Structure Investigation and Parametrization of Biologically Relevant Iron–Sulfur Clusters, Journal of Chemical Information and Modeling 54 (2014) 613-620.

[4] W.L. Jorgensen, J. Chandreskhar, J.D. Madura, R.W. Imprey, M.L. Klein, Comparison of simple potential functions for simulating liquid water, J. Chem. Phys. 79 (1983) 926-935.

[5] D.A. Case, Ben-Shalom, I.Y., Brozell, S.R., Cerutti, D.S., Cheatham, T.E. III, Cruzeiro, W.D. V., Darden, T.A., Duke, R.E., Gilson, M.K., Gohlke, H., Goetz, A.W., Greene, D., Harris, R., Homeyer, N., Huang, Y., Izadi, S., Kovalenko, A., Kurtzman T, Lee, T.S., LeGrand, S., Li, P., Lin, C., Liu, J., Luchko, T., Luo, R., Mermelstein, D.J., Merz,. K.M., Miao, Y., Monard, G., Nguyen, C., Nguyen, H., Omelyan, I., Onufriev, A., Pan, F., Qi, R., Roe, R. D., Roitberg, A., Sagui, C., Schott-Verdugo, S., Shen, J., Simmerling, C.L., Smith, J., SalomonFerrer, R., Swails, J., Walker, R.C., Wang, J., Wei, H., Wol,f R.M., Wu, X., Xiao, L., York, D.M. and Kollman, P.A., AMBER 2018, University of California, San Francisco, 2018.

[6] T. Darden, D. York, L. Pedersen, Particle mesh Ewald: an N log(N) method for Ewald sums in large systems, J. Chem. Phys. 98 (1993) 10089-10092.

[7] T.E. Cheatham, J.L. Miller, T. Fox, T.A. Darden, P.A. Kollman, Molecular-Dynamics simulations on solvated biomolecular systems - the particle mesh Ewald eethod leads to stable trajectories of DNA, RNA, and proteins, J. Am. Chem. Soc. 117 (1995) 4193-4194.

[8] J.P. Ryckaert, G. Ciccotti, B.H.J. C., Numerical integration of the cartesian equations of motion of a system with constraints: molecular dynamics of n-alkanes, J. Comput. Phys. 23 (1977) 327-341.

[9] R.J. Loncharich, B.R. Brooks, R.W. Pastor, Langevin dynamics of peptides: the frictional dependence of isomerization rates of N-acetylalanyl-N'-methylamide, Biopolymers 32 (1992) 523-535.

[10] H.J.C. Berendsen, J.P.M. Postma, W.F. van Gunsteren, A. DiNola, J.R. Haak, Molecular dynamics with coupling to an external bath, The Journal of Chemical Physics 81 (1984) 3684-3690.

[11] D.R. Roe, T.E. Cheatham, PTRAJ and CPPTRAJ: Software for processing and analysis of molecular dynamics trajectory Data, J. Chem. Theory Comput. 9 (2013) 3084-3095.

[12] H. Flyvbjerg, H.G. Petersen, Error estimates on averages of correlated data, The Journal of Chemical Physics 91 (1989) 461-466.

[13] W. Yang, R. Bitetti-Putzer, M. Karplus, Free energy simulations: use of reverse cumulative averaging to determine the equilibrated region and the time required for convergence, J Chem Phys 120 (2004) 2618-2628.

[14] Schrodinger, LLC, The PyMOL Molecular Graphics System, Version 1.8, in, 2015.

[15] W. Humphrey, Dalke, A., and Schulten, K., VMD - Visual Molecular Dynamics., Journal of Molecular Graphics 14 (1996) 33-38.

[16] J.Y. Shao, S.W. Tanner, N. Thompson, T.E. Cheatham, Clustering molecular dynamics trajectories: 1. Characterizing the performance of different clustering algorithms, J. Chem. Theory Comput. 3 (2007) 2312-2334.

[17] G. Kokic, A. Chernev, D. Tegunov, C. Dienemann, H. Urlaub, P. Cramer, Structural basis of TFIIH activation for nucleotide excision repair, Nat Commun 10 (2019) 2885.

[18] H. Khandelia, Y.N. Kaznessis, Cation-pi interactions stabilize the structure of the antimicrobial peptide indolicidin near membranes: molecular dynamics simulations, J Phys Chem B 111 (2007) 242-250.
